# Advanced Epoxy Polymer Composite Design: Maximizing Fracture Toughness and Piezoelectric Effect with Core–Shell Rubber and Barium Titanate Particles

**DOI:** 10.1021/acsomega.5c03542

**Published:** 2025-09-26

**Authors:** Miray Yasar, Berran Sanay, Cormac Duffy, Neal Murphy, Barry Burns, Alojz Ivankovic

**Affiliations:** † 8797 University College Dublin, School of Mechanical and Materials Engineering, Dublin 4, Ireland; ‡ I-Form Advanced Manufacturing Research Centre, Dublin, Dublin 4, Ireland; § 111803 Henkel Ireland Operations & Research Ltd., Dublin, D24 YH42, Ireland

## Abstract

Epoxy resins, when cured are important thermoset polymers which possess exceptional bonding strength, versatility, and resistance, making them indispensable in various industries ranging from construction to electronics. This study aims to investigate epoxy-based polymer composites with varying core–shell rubber (CSR) fractions (0–5–10–15–25 wt %) with a fixed weight percentage (20 wt %) of barium titanate (BaTiO_3_) and tailor the thermal, mechanical and piezoelectric behavior of the resulting materials. Tensile, single lap shear and T-Peel strength tests were conducted to evaluate the effects of CSR and BaTiO_3_ additives on the mechanical properties of the composites. Fracture toughness and fracture energy measurements were performed using the single-edge notched bend test to assess the crack resistance of the composites. The thermal-mechanical properties of the composites were analyzed using a dynamic mechanical analyzer. A voltage output test was performed to evaluate the piezoelectric properties of the composites. The results demonstrated that with 5 wt % CSR incorporation, the composite exhibited a remarkable increase in fracture energy, increasing from 465 to 2213 J/m^2^, and with 15 wt % CSR addition, the highest open circuit voltage output of 1.85 V was obtained.

## Graphical abstract

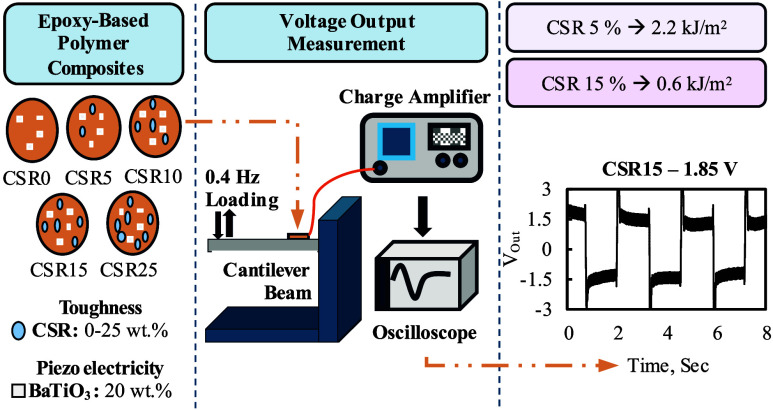

## 1 Introduction

Epoxy resin, a form of thermosetting polymer with a strong network of chemical bonds, is extensively utilized in producing composite materials reinforced with fibers and as structural adhesives and coatings. They typically have high modulus, remarkable strength, minimal deformation over time (low creep), and exceptional resistance to elevated temperatures.^1,2^ Nonetheless, the highly interconnected structure of epoxy resin also presents certain limitations, such as inherent low resistance to fracture and poor fracture toughness.^2,3^ It becomes imperative to enhance the fracture toughness of these materials while minimizing any significant compromise to their rigidity and strength.

The incorporation of additional components has been discovered to be a practical approach for enhancing the toughness of epoxy materials. Resistance to crack growth and failure is improved by adding various modifiers, such as reactive rubber particles,^3,4^ thermoplastic polymers,^5,6^ thermoplastic particles,^7^ and rigid additives such as nanosilica particles,^8^ block copolymers, and dendritic polymers have shown the ability to enhance the fracture toughness of epoxy matrix.^9^ Rubber particles have gained particular attention among these modifiers due to their exceptional ability to toughen epoxy polymers.^5,6^ Blending epoxy polymers with butadiene-acrylonitrile-based liquid rubbers, such as carboxyl-terminated butadiene–acrylonitrile^10^ and other rubbers, such as amino-terminated^11^ and vinyl-terminated rubbers^12^ were extensively investigated.

The incorporation of the second generation of rubber modifiers, featuring structured core–shell particles with a rubbery core and a thin plastic shell, has further provided advancements in toughening agents.^13^ This core–shell structure offers a synergistic combination of toughness and stiffness, thereby effectively strengthening the mechanical properties of epoxy matrices. Unlike liquid rubbers, core–shell rubber (CSR) particles are compatible with epoxy polymers, reducing the possibility of incomplete phase separation. The structure of the core–shell rubber allows compatibility due to the plastic shell and the rubber core increasing the toughness.^9^ The soft core is generally butadiene rubber, poly­(butyl acrylate), styrene–butadiene rubber or polysiloxane, and the harder shell is commonly poly­(methyl methacrylate).^14^ The role of core–shell rubber is to reduce hydrostatic tension and start the ductile shear yielding mechanism by creating cavities.^9^ Becu et al.^15^ studied the fracture behavior of core–shell rubber toughening bisphenol A epoxy matrix. Fracture toughness and impact strength increased by the addition of CSR up to 24 vol %, and the crack growth rate was reduced. Chen et al.^16^ modified an epoxy resin by the addition of polysiloxane CSR, which increased the fracture toughness of the epoxy matrix significantly at room temperature and cryogenic temperature conditions. Quan and Ivankovic^1^ investigated the effect of core–shell rubber nanoparticles on the mechanical properties and fracture toughness of an epoxy matrix. They obtained an increment in fracture energy from 343 to 2671 J/m^2^ by 30 vol % CSR addition. Quan et al.^17^ also studied the synergetic effect of CSR and silica nanoparticles on epoxy in terms of its fracture toughness, glass transition temperature, tensile modulus, and yield strength. They obtained maximum fracture toughness with 7 vol % CSR and 4 vol % silica.

In addition to mechanical reinforcement, functional additives such as piezoelectric materials are crucial for imparting piezoelectric properties to epoxy composites. Piezoelectric ceramics such as lead zirconate titanate^18^ with the highest piezoelectric properties, barium titanate (BaTiO_3_)^19–22^ aluminum nitride,^23^ and other inorganic compounds such as zinc oxide,^24^ perovskites such as lithium tantalate,^25^ salts such as potassium sodium tartrate,^26^ crystals such as quartz^27^ and piezoelectric polymers such as polyvinylidene fluoride, polyacrylonitrile, polyaniline, were incorporated into the various polymer materials to prepare piezoelectric polymer composites. Epoxy is chemically stable and durable in a cured state, which can be used for piezoelectric device design. Piezoelectric lead-free perovskite-type barium titanate is a balanced option regarding environmental safety and a high piezoelectric coefficient.^28,29^

In recent years, the development of ternary and quaternary epoxy composite systems incorporating CSR and an additional filler has garnered growing attention due to their potential to simultaneously enhance mechanical properties and introduce multifunctionality. For example, Chen et al.^30^ investigated the effects of incorporating κ-aluminum oxide (κ-Al_2_O_3_) nanosheets and carboxyl-terminated butadiene-acrylonitrile (CTBN) liquid rubber into an epoxy resin matrix. They reported that the addition of κ-Al_2_O_3_ significantly improved the thermal and mechanical properties of the composites. Notably, at 5 phr κ-Al_2_O_3_ content, the nanocomposite exhibited a 21 °C increase in glass transition temperature and enhancements of approximately 22% in tensile modulus and 50% in elongation at break compared to the CTBN/epoxy blend. In another study, Ma et al.^31^ developed an analytical model to predict the fracture toughness of epoxy resins modified with poly­(ether imide) (PEI) and carbon nanotubes (CNTs). They showed that at low PEI contents, shear banding dominated, whereas at higher contents (≥20 wt %), crack bridging became the primary toughening mechanism. In ternary CNT/PEI/epoxy systems, improved CNT dispersion led to a synergistic enhancement in toughness. Moreover, Zhu et al.^32^ prepared hybrid epoxy nanocomposites by incorporating zinc oxide-functionalized multiwalled carbon nanotubes (ZnO-MWCNTs) and core–shell rubber (CSR) into an epoxy matrix to achieve simultaneous improvement in mechanical properties. The incorporation of 1.5 wt % ZnO-MWCNTs provided high stiffness and strong interfacial adhesion, while the addition of 3 wt % CSR introduced a soft phase capable of inducing cavitation and shear banding. As a result, the ternary system exhibited a significant enhancement in fracture toughness (from 0.82 MPa·m^1/2^ for neat epoxy to 1.46 MPa·m^1/2^) without compromising the glass transition temperature. The observed toughening was attributed to a synergistic combination of mechanisms, including CSR-induced matrix shear banding, MWCNT fracture, and crack deflection. Likewise, Bajpai et al.^33^ investigated the effects of CSR, MWCNTs, and SiO_2_ nanoparticles on a bisphenol A based epoxy system. The highest enhancement in fracture toughness (1.75 MPa·m^1/2^) and fracture energy (1010 J/m^2^) was achieved with a ternary formulation containing 5 wt % CSR and 10 wt % SiO_2_, corresponding to increases of 207% and 910%, respectively, compared to neat epoxy. The electrical percolation threshold was reached at 0.075 wt % MWCNT, with conductivity increasing to 0.00163 S/m at 0.1 wt %. While CSR decreased tensile strength and modulus, these were effectively recovered through the addition of rigid SiO_2_ nanoparticles. This hybrid toughening approach demonstrated a promising balance of toughness, conductivity, and mechanical integrity for high-performance applications.

Despite numerous studies on epoxy-based composites, there remains a gap in understanding how CSR and BaTiO_3_ collectively affect the multifunctional performance. This study systematically explores the impact of varying core–shell rubber concentrations while maintaining a constant barium titanate content. It aims to investigate the characteristics of epoxy-based piezoelectric polymer composites in terms of their mechanical properties (e.g., tensile strength, T-peel and lap shear strength), fracture toughness, thermal behavior (e.g., glass transition temperature), structural and morphological features, and piezoelectric response. To the best of our knowledge, this is the first systematic investigation addressing the combined effects of CSR and BaTiO_3_ on these multifunctional properties in a single epoxy-based composite system. The findings aim to bridge crucial knowledge gaps and support the development of multifunctional composites for high-performance applications in fields such as aerospace and electronics. The toughening mechanism of CSR is primarily attributed to localized plastic deformation and cavitation-induced crack deflection, which dissipates energy and delays crack propagation. Meanwhile, BaTiO_3_, as a ferroelectric ceramic, contributes to piezoelectric response by generating electrical charges under mechanical stress, enabling the voltage generation within the polymer matrix.

## 2 Experimental Section

### 2.1 Materials

In this research, a standard diglycidylether of bisphenol A (DGEBA) epoxy (Epon828, HEXION) with an epoxy equivalent molecular weight of between 185 g/eq and 192 g/eq was used. A combination of Amicure CG 1200 (DICY – Evonik Hanse), Dyhard (UR700, AlzChem Group AG) and Ajicure PN-23 (Ajinomoto Fine-Techno Co. Inc.) were used as an accelerator and curing agents. The core–shell rubber (CSR) particles, consisting of a polybutadiene core and a poly­(methyl methacrylate) (PMMA) shell, were supplied by Kaneka as a master batch (Kane Ace MX-154), containing 40 wt % of CSR particles (average diameter of 100 nm^34^) dispersed in a DGEBA resin. Micron size (0.5–1 μm) tetragonal crystalline structure BaTiO_3_ (BT) was obtained from abcr Gute Chemie. The BT content was fixed at 20 wt % across all formulations. This specific concentration was selected based on our previous study,^21^ in which epoxy composites containing only BT (without CSR) were investigated using the same epoxy resin but different curing systems. That study demonstrated that 20 wt % BT provided the highest piezoelectric output among all tested formulations, while maintaining processability. Although the formulation used in this study differs from the previous one, the same BT content was maintained to investigate the influence of core–shell rubber (CSR) in a system that had previously demonstrated effective piezoelectric behavior. The current focus is to examine how CSR incorporation affects the mechanical performance, particularly toughness, and the piezoelectric response of the polymer composites.

The formulation allowed curing at 100 °C, a temperature below the Curie temperature, to preserve the tetragonal structure of the BT particles and prevent any alteration of their piezoelectric properties. Silver-loaded epoxy adhesive (MG 8331) from MG Chemicals was used to secure the wiring for the samples prepared for piezoelectric measurements. Loctite 454 cyanoacrylate instant adhesive was used to bond the piezoelectric material onto the cantilever beam, provided by Henkel, Ireland.

Table 1 tabulates the basic compositions of the specimens, which are named according to CSR content. Each combination includes 20 wt % BT and a certain ratio of the epoxy curative mixture.

**Table 1:** Formulations of the Epoxy-Based Polymer Composites Containing 20 wt % BT and Varying Amounts of CSR (0–25 wt %)

name	Epon 828, %	CSR, %	BT, %	Amicure CG 1200, %	Dyhard UR700, %	Ajicure PN23, %
CSR0 (ref)	67.0	0	20	5.6	1.4	6.0
CSR5	62.7	5	20	5.3	1.3	5.7
CSR10	58.6	10	20	4.9	1.2	5.3
CSR15	54.4	15	20	4.5	1.2	4.9
CSR25	46.0	25	20	3.9	1.0	4.1

### 2.2 Methodology

#### 2.2.1 Sample Preparation

The CSR modified epoxy resin and BT particles were mixed using a high-speed mechanical stirring method with a Eurostar 20 high-speed digital mixer, operating at 2000 rpm for 30 min and 5000 rpm for 5 min at 60 °C. The mixture was then cooled down to room temperature. Curatives were added and mixed using a mechanical mixer at 2000 rpm for 30 min. Subsequently, an ESCO vacuum shear mixer was employed to enhance dispersion and eliminate the air bubbles. Different formulations with varying CSR contents were explored, ranging from 0 to 25 wt %, as outlined in Table 1.

For the mechanical measurements, the mixture was poured into molds which were precoated with a release agent (Frekote 700NC, Henkel). Then, the samples were cured at 100 °C for 2 h.

For piezoelectric measurements, sample preparation began with sticking copper tape (serving as a bottom electrode) onto a polyester film (Hifi-SRF122). A single layer of the epoxy-based polymer composite was applied onto the copper tape using a paintbrush and partially cured in a preheated oven at 100 °C for 20 min. The epoxy layer thickness ranged between 0.200–0.350 mm. The samples were intentionally left partially cured to allow chain movement during the subsequent poling process.

After partial curing, the polymer layer was carefully peeled from the polyester film, and the second copper tape was adhered to the surface of the epoxy layer to serve as the top electrode. The poling process was carried out using a contact poling setup consisting of:

(1) A high voltage power supply (Branderburg Alpha Series III, 30 kV-1.5 mA),
(2) An in-house poling unit,
(3) A silicone oil bath (heated to 100 °C) and
(4) A hot plate.

The sample was immersed in a silicone oil bath, which served two primary purposes: (1) enabling high temperature poling, and (2) providing electrical insulation to prevent dielectric breakdown and electrical arcing during the application of high voltage. A voltage of 50 V/μm was applied through the thickness of the sample for 120 min while submerged in the oil bath maintained at 100 °C. The sample was positioned between a spring-loaded steel rod connected to the high voltage terminal and the grounded bottom aluminum plate.

Upon completion of the simultaneous poling and curing process at the specified cure temperature and duration, any residual silicone oil was cleaned using isopropanol. Electrical wires were connected to both copper electrodes using a silver-loaded epoxy adhesive, which was cured at room temperature for 24 h. The samples were then cut from the edges of the copper tapes and encapsulated with Kapton tape to prevent environmental and electrical interference. A schematic representation of the complete preparation and poling setup is provided in Figure 1.

**Figure 1:**
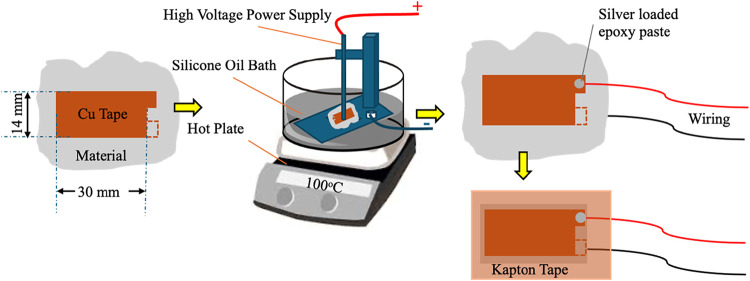
Preparation of epoxy-based polymer composite films for voltage output test.

### 2.3 Analyses

#### 2.3.1 Mechanical Properties

The mechanical properties of the bulk polymer were characterized through a series of tests.

The single-lap shear test was conducted in accordance with ASTM D1002, using a Zwick/Roell Proline Z005 universal testing machine with a crosshead speed of 1 mm/min at room temperature. The surface of the steel panels (Q-Panel, RS-14) with dimensions of 25 × 102 mm^2^ and a thickness of 1.6 mm (Figure 2) was cleaned with isopropanol and clean cloths. A specific apparatus was used to ensure a uniform thickness of 0.2 mm and proper alignment. The polymer composite was applied to a 12.7 × 25 mm^2^ bonding area and cured at 100 °C for 2 h using a fan-assisted oven.

**Figure 2:**
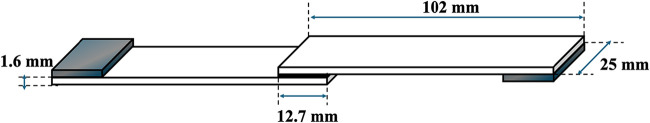
Schematic of the single-lap joint test.

T-peel test specimens were prepared in accordance with ISO 11339:2022. They were tested using a Zwick/Roell Proline Z005 universal testing machine with a crosshead speed of 100 mm/min at room temperature. Test specimens were prepared individually. Aluminum test panels were used with dimensions of 25 mm width × 200 mm length and 0.8 mm thickness (Figure 3). The bonding surface of the aluminum test specimens was first abraded using P240 grit sandpaper and then cleaned with isopropanol. The polymer composite was applied to a 150 × 25 mm^2^ bonding area with a thickness of 0.3 mm. Curing was carried out at 100 °C for 2 h using a fan-assisted oven.

**Figure 3:**
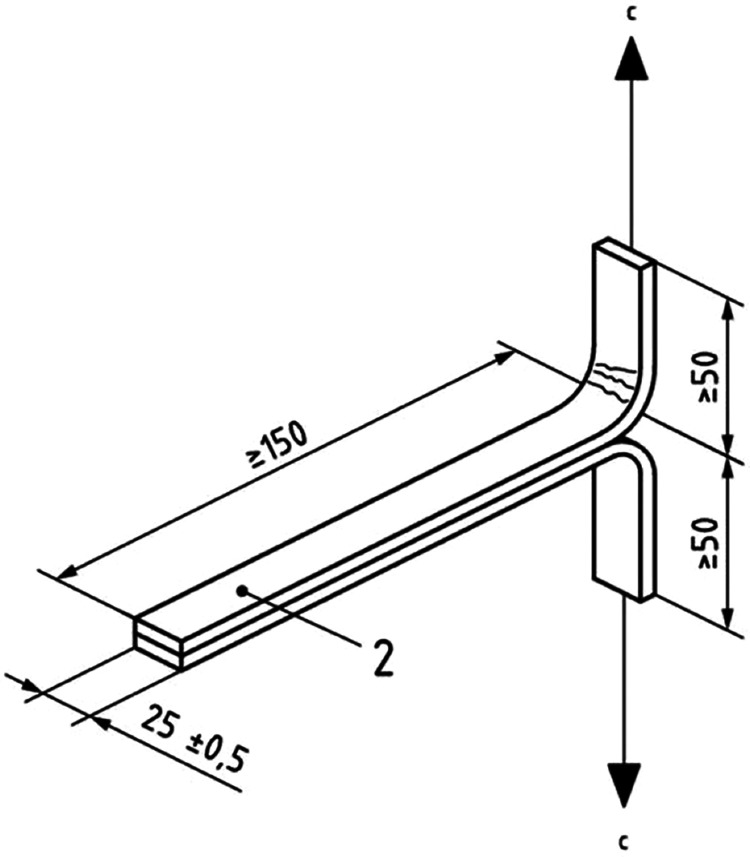
Schematic of the specimen of the T-peel test.

The true adhesive fracture energy was calculated using the freely available ICPeel Software from Imperial College London,^35^ which provides a full analytical solution. The theoretical basis of the method is detailed in the study of Kinloch et al.^36^ The peel arms (aluminum test panels) were tested after the T-peel test. A steady peel rate of 1 mm/min was maintained. The gauge length of the peel arm sample was 50 ± 0.5 mm.

The interlaminar fracture energy, G_C_, for each peel arm, was calculated using eq 1.

1 $$G_{C}=G-G_{P}\left(R_{0}\right)$$

where *G* is the total energy used for peel fracture and *G*_P_ (*R*_0_) is the plastic bending energy associated with deforming the peel arm to a minimum radius of curvature *R*_0_. *G* was calculated through the peel strength of the adhesive (peel force per unit width), and *G*_P_ was calculated using the software ICPeel with the required data received from peel arm tensile stress–strain behavior. This data was fitted to a linear power law mathematical model following the ESIS TC4 Peel Test protocol.

The tensile strength, Young’s modulus and elongation at break values were characterized in accordance with ISO 527 Model 1BA. Dumbbell specimens with a gauge length of 25 mm were prepared from the bulk epoxy polymer composites. A Zwick/Roell Proline Z005 universal testing machine was used with a crosshead speed of 0.5 mm/min at a room temperature of 22 °C. The changes in gauge length and width of the sample were measured using a noncontact video extensometer. The maximum tensile stress for each sample was recorded, and the elastic modulus, *E*, was calculated between strains of 0.05 and 0.25%. At least six replicate samples were tested for each formulation.

#### 2.3.2 Fracture Toughness

A single-edge notched three-point bending (3PB) test was used to measure the fracture toughness (*K*_IC_) and fracture energy (*G*_IC_) of the polymer composites in accordance with ASTM D5045-99 standard.^37^ A precrack was introduced by tapping a chilled razor blade into the bottom of the V-shape notch. The specimens, with dimensions of 52.8 mm length, 12 mm width (*W*), 6 mm thickness (*B*), and 48 mm span (*S*), were used. Testing was performed at room temperature (22 °C) with a constant displacement rate of 1 mm/min using a Zwick/Roell Proline Z005 universal testing machine. The lengths of the precracks were measured with an Ash Inspex HD 1080p Digital Microscope.

A brittle fracture occurred in every specimen, and the stress intensity factor, *K*_IC_, was calculated using eq 2

2 $$K_{\mathrm{IC}}=\frac{P_{c}}{{\mathrm{BW}}^{1/2}}f\left(\varepsilon \right)$$

where

3 $$f\left(\varepsilon \right)=\frac{6\varepsilon^{1/2}\left\{1.99-\varepsilon \left(1-\varepsilon \right)\left(2.15-3.99\varepsilon +2.7\varepsilon^{2}\right)\right\}}{(1+2\varepsilon ){\left(1-\varepsilon \right)}^{3/2}}$$

4 $$\varepsilon =\frac{a}{W}$$

*P*_c_ is the maximum load; *B* (mm), *a* (mm), and *W* (mm) are the thickness, the precrack length and the width of the specimen, respectively.

The value of the fracture energy, *G*_IC_, was calculated using *K*_IC_ and *E* with eq 5.

5 $$G_{\mathrm{IC}}=\frac{K_{\mathrm{IC}}^{2}}{E}\left(1-v^{2}\right)$$

where *E* is the modulus of elasticity (MPa) obtained from the tensile tests, and *v* is the Poisson’s ratio of the polymer.

Fracture energy can be also calculated using the *J*-integral, which is a measure of the energy release rate during crack propagation in a material. It represents the energy available for crack growth per unit of crack surface area. For linear elastic materials, the *J*-integral is equivalent to the fracture energy, *G*_IC_.

The energy-based calculation of *G*_IC_ using the area under the load–displacement curve is an experimental representation of the *J*-integral. The fracture energy is calculated as

6 $$G_{\mathrm{IC}}\left(J\right)=\frac{U}{\mathrm{hW}⌀}$$

where *U* is the total energy absorbed during fracture, represented by the area under the load–displacement curve, *h* is the specimen thickness (mm), *W* is the specimen width (mm), ⌀ is the correction factor for geometry obtained from ASTM D5045-99.

This approach aligns with the *J*-integral formulation, which evaluates the total energy dissipation for a crack in a loaded body.

#### 2.3.3 Dynamic Mechanical Thermal Analyses (DMTA)

DMTA was performed to evaluate the thermo-mechanical properties of the cured epoxy-based polymer bulk specimens. The glass transition temperature (from the peak value of the loss factor, tan δ) and the storage modulus (*E*’) were determined using a DMTA 242 E Artemis (Netzsch, Germany). The tests were carried out on samples with dimensions of 10 mm in length, 6 mm in width and 1.3 mm in thickness in tension mode at 1 Hz. The temperature was increased from 30 to 200 °C with a heating rate of 3 °C/min and a constant amplitude of 10 μm. DMTA specimens were produced by pouring the epoxy-based polymer composite into a precoated mold. The samples were cured at 100 °C for 2 h in a fan-assisted oven. The surface of the specimens was polished with P600 grit sandpaper until a thickness of 1.3 mm was reached.

The cross-link densities, *v*_*XL*_ (mol/m^3^), of the polymer composites were calculated using the Flory theory^38^ as given in eq 7.

7 $$v_{\mathrm{XL}}=\frac{E^{\prime }}{3\mathrm{RT}}$$

where *E*’ is the storage modulus, *R* is the gas constant (equal to 8.314 J.K^–1^·mol^–1^), *T* is the absolute temperature at *T*_g_+50 °C, and *E*’ is the storage modulus at a temperature of *T*_g_+50 °C.

The fraction of constrained polymer chains in epoxy-based nanocomposites was quantitatively calculated from the DMTA data using the method employed by Poornima Vijayan et al.^39^ This method is based on the peak height of the tan δ curve, which reflects the amount of energy dissipation due to segmental motion of polymer chains during the glass transition.

In linear viscoelastic materials, the energy loss fraction (*W*) at the tan δ peak is calculated using eq 8.

8 $$W=\frac{\pi \cdot \tan\delta }{\pi \cdot \tan\delta +1}$$

To determine the constrained volume fraction of the constrained region (*C*), the following relation is used

9 $$C=\frac{\left(1-C_{0}\right)\cdot W}{W_{0}}$$

Here, *W*_0_ and *C*_0_ correspond to the energy loss fraction and the constrained region fraction of the reference system, respectively. In most studies, *C*_0_ = 0 is assumed for the reference sample, representing a fully amorphous polymer with no additional confinement effects.

#### 2.3.4 Toughening Mechanism

The morphology of the fracture surfaces was studied by a Hitachi TM4000 Tabletop scanning electron microscope (with an accelerating voltage of 5 kV), a Hitachi Regulus 8230 SEM for higher magnification and an Ash Inspex HD 1080p Digital Microscope. The fracture surfaces of single-lap shear fracture test specimens and three-point bending test specimens were analyzed. Samples were gold sputtered to increase the conductivity and prevent charging for SEM analyses. Images were taken near the notch tips of three-point bending samples.

#### 2.3.5 X-ray Diffraction

XRD analysis was performed using a Siemens D500 X-ray Diffractometer at angles of 10–55°, with a 0.02 step size (degree) and a 1 s/step dwell time per step.

#### 2.3.6 Voltage Output Tests

The voltage output of the samples was measured through an open circuit setup consisting of a function generator, a linear actuator, a stainless steel cantilever beam (210 mm × 25.15 mm × 1 mm), strain gauges (Vishay, EA-06-062AQ-350), a signal conditioning amplifier (Vishay 2310B), a charge amplifier (Kistler Type 5019A), and an oscilloscope (TiePie Handyscope HS3). The piezoelectric sample was placed onto the cantilever beam using a cyanoacrylate instant adhesive. A half-bridge strain gauge circuit was connected on the underside of the cantilever to measure deformation. The strain signals were processed by the signal conditioning amplifier and visualized via the oscilloscope.

Mechanical loading was applied using a linear actuator, which was driven by the function generator producing a square waveform at a frequency of 0.4 Hz, as shown in Figure 4a. This cyclic loading induced periodic bending of the cantilever beam, enabling the piezoelectric composite to generate a measurable voltage output. The sample was connected to the charge amplifier input through a coaxial piezoelectric cable (Kistler, Product Type: 1601B1), and the amplified signal was monitored using the oscilloscope.

**Figure 4:**
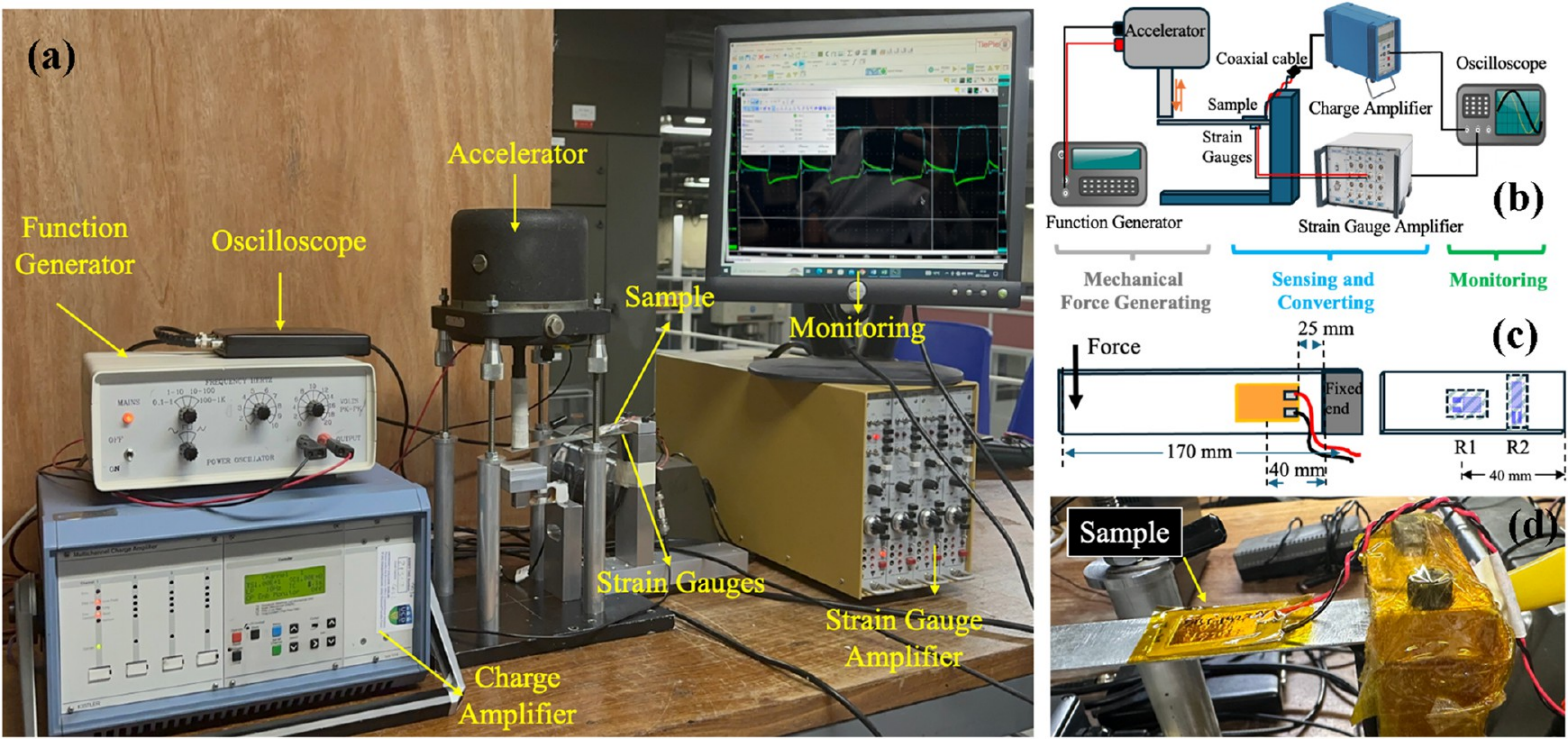
(a) Voltage output measurement setup, (b) schematic explanation of the voltage output measurement setup, (c) sample and strain gauge locations on a cantilever beam, (d) photo of sample attached to the cantilever beam.

A schematic overview of the experimental configuration is shown in Figure 4b. Figure 4c shows the cantilever beam configuration, with the top view on the left illustrating the placement of the piezoelectric sample, and the bottom view on the right displaying the locations of two strain gauges (R1 and R2) mounted beneath the beam. R1 is aligned directly beneath the sample along the beam axis, while R2 is oriented transversely, serving to compensate for temperature variations. Figure 4d shows a photograph of the sample positioned on the cantilever beam during testing.

To calculate the voltage output generated by a material, the charge (q) was first calculated using eq 10.

10 q=transducersensitivity×scale×outputvoltage

where transducer sensitivity and scale are parameters of the charge amplifier, with the units of pC/mechanical unit and mechanical unit/volt, respectively. The output voltage is monitored via computer software connected to the oscilloscope. It should be noted that this output voltage does not represent the actual voltage across the material. Rather, it is an amplified signal used to calculate the generated charge. The true output voltage across the material (*V*_c_) is then derived from the calculated charge and the material’s capacitance (*C*_s_).

The capacitance C_s_ was measured using Wayne Kerr Automatic LCR Meter 4225 at 1 kHz, and the actual voltage across the material was calculated using eq 11.

11 $$V_{c}=\frac{q}{C_{s}}$$

The strain value was measured using a half-bridge strain gauge configuration (one transversely arranged, one longitudinally arranged) under the stainless-steel cantilever beam, as seen in Figure 4c.

The output signal *V*_o_ of the measuring bridge is referenced to the feed voltage *V*_s_. The sensitivity GF of the strain gauge enables the strain ε to be calculated for the half-bridge as follows (eqs 12–14).

12 $$V_{0}=K\frac{\Delta R}{R}V_{s}$$

where *K* is 1/2 for a half-bridge.

13 $$\mathrm{GF}=\frac{1}{\varepsilon }\frac{\Delta R}{R}$$

14 $$\varepsilon =\frac{V_{0}}{V_{s}}\frac{2}{\mathrm{GF}}$$

where GF is 2.085 ± 0.5.

Referring to the study of Sirohi and Chopra,^40^ the piezoelectric coefficient d_31_ is calculated by using eq 15.

15 $$d_{31}=\frac{q\left(pC\right)}{\varepsilon \cdot E\cdot L_{c}\cdot b_{c}\left(N\right)}$$

where *E* (MPa) is the Young’s modulus of the piezo material, and *L*_c_ (mm) and *b*_c_ (mm) are the length and width of the piezo film.

## 3 Results and Discussion

### 3.1 Mechanical Properties

#### 3.1.1 Lap-Shear Strength

The single-lap shear test was performed to determine the maximum load that the unit bonding area of the polymer composites can withstand.^41^ Q-Panel, RS-14 steel panels were used as the substrate. Figure 5 shows the lap-shear strength of the epoxy composites with varying CSR amounts and fixed BT, as measured in a single-lap shear test. The lap-shear strength increased from 15.7 MPa (CSR0) to 22.5 MPa (CSR5) and peaked at 23.6 MPa with CSR10. Beyond this point, the values slightly decreased.

**Figure 5:**
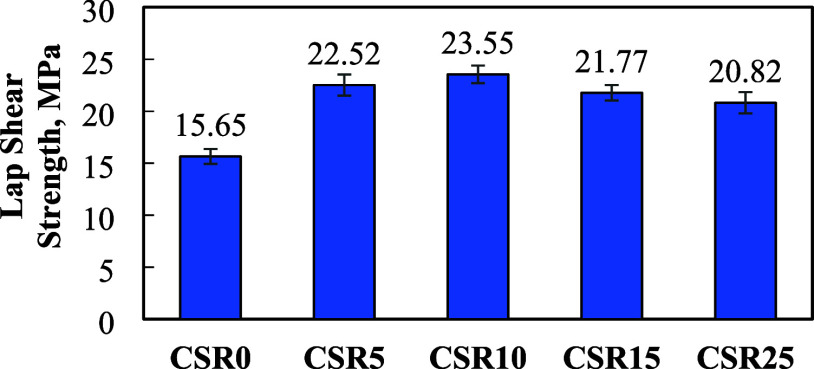
Lap-shear strength of the epoxy-based polymer composites containing 20 wt % BT and varying amounts of CSR (0–25 wt %), bonded to steel substrates.

Adhesive failure was predominantly observed in the samples, as shown through the fracture surface analysis of the lap-shear specimens (Figure 6a). Further examination using SEM (Figure 6b) provided insights into the morphology of the fracture surfaces, with the analyzed areas highlighted by red dashed lines. In this study, BT was used in micron-sized form, while CSR particles were nanoscale. As a result, CSR particles could not be observed in the same SEM images where BT particles were visible. A more detailed analysis of toughening mechanisms, including CSR-related morphology, is presented in Section 3.3, based on higher magnification SEM images of the fracture surfaces from 3PB specimens.

**Figure 6:**
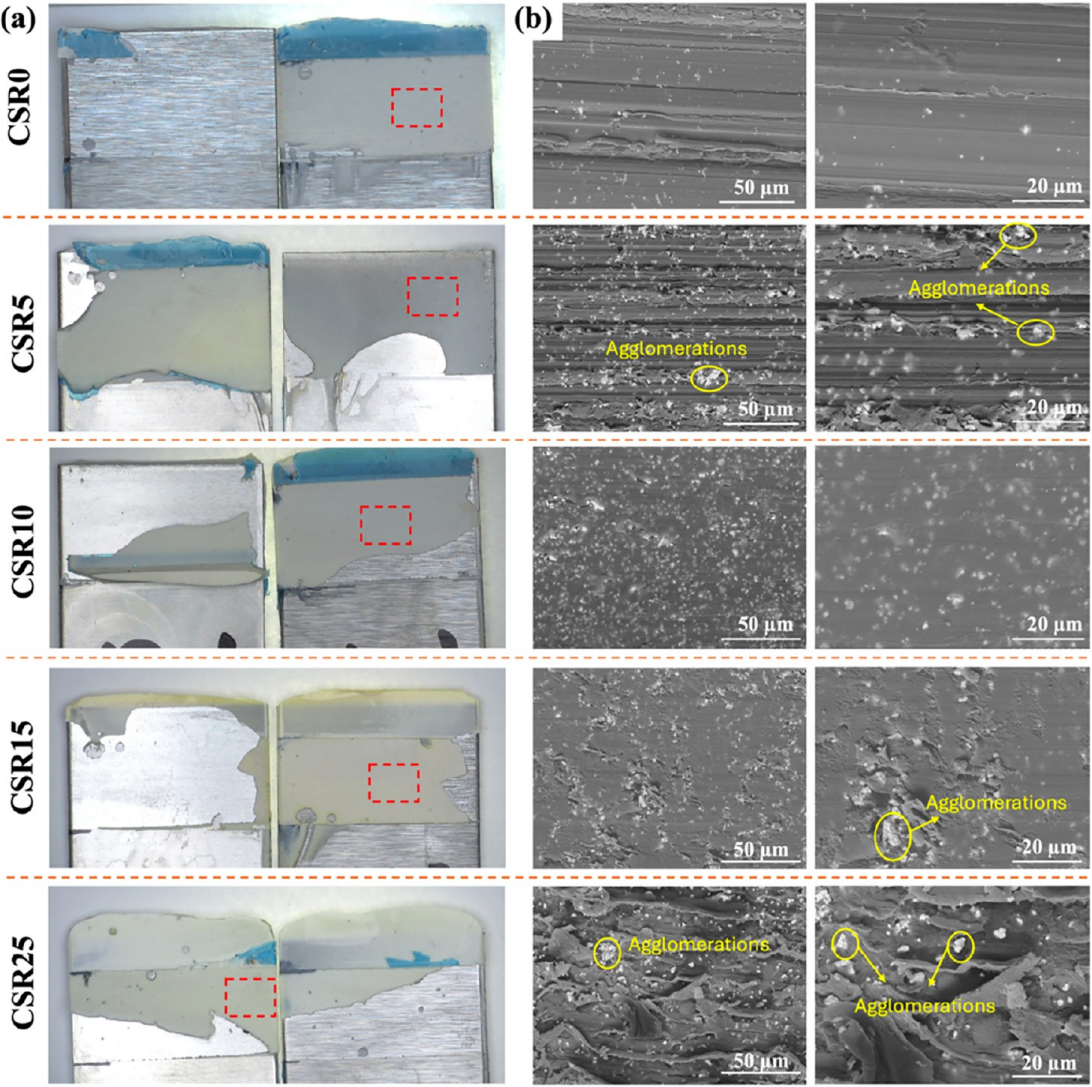
Fracture surface of the single-lap shear test specimens containing 20 wt % BT and varying amounts of CSR (0–25 wt %) by (a) optical microscopy and (b) SEM micrographs. Scale bars and magnifications: 50 μm/×1800 and 20 μm/×4000.

SEM analysis showed a relatively smooth surface for the CSR0 sample, while rougher features became evident as the CSR content increased, starting at CSR5 as shown in Figure 6b. These rough features on the fracture surfaces may be attributed to the deformation of CSR particles under combined shear and peel stresses during fracture.^42^ Similar rough surface characteristics were reported by Baek et al.,^42^ who observed deformation-induced roughness with the incorporation of CSR particles composed of poly­(methyl methacrylate) (PMMA) and butadiene rubber. They reported a lap-shear strength of 22 MPa at 14.8 wt % CSR content at 20 °C.

Since the BaTiO_3_ content was constant across all formulations, the observed variation in lap shear strength primarily reflected the effect of CSR concentration. The relatively consistent strength values across different CSR levels suggested that the BT particles were generally well dispersed within the matrix and contributed to the mechanical performance, although some agglomerations were observed, as indicated in Figure 6b. The formation of these agglomerates was related to the increased viscosity at higher CSR contents, which affected mixing and dispersion during processing.

These results indicate that both the distribution of BT particles and the toughening effect of CSR influenced the lap shear strength, although a direct interaction between CSR and BT could not be confirmed due to differences in magnification across SEM analyses. Their combined influence on composite morphology and mechanical behavior is further examined through the fracture surface analysis of the 3PB test specimens (Section 3.3).

#### 3.1.2 T-peel Test

T-peel strength and adhesive fracture energy were investigated, and the results are shown in Figure 7a,7b, respectively. The T-peel test measures the tensile and peeling forces perpendicular to the bond line of the adhesive-bonded joint.^43^ Aluminum panels were used as the substrate.

**Figure 7:**
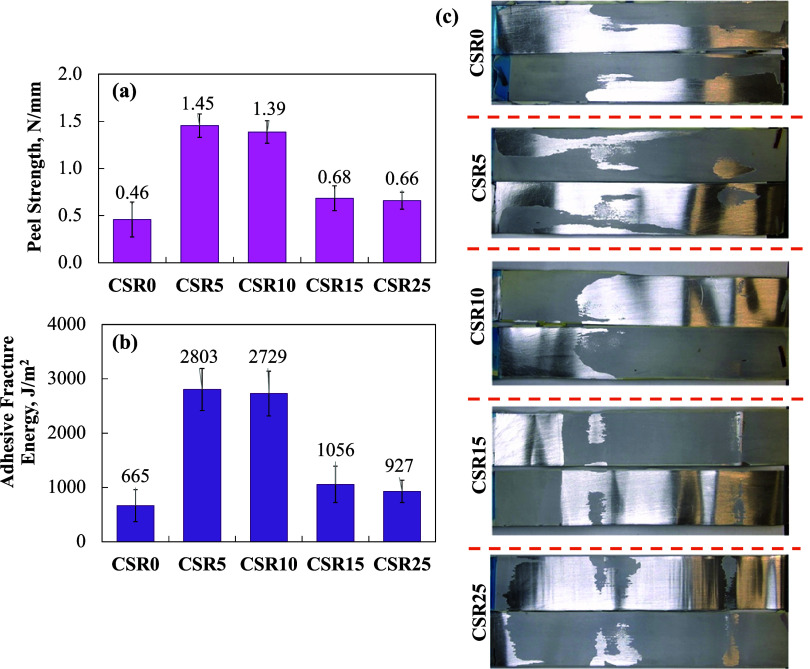
(a) T-peel strength, (b) adhesive fracture energy, (c) T-peel surfaces of the epoxy-based polymer composites containing 20 wt % BT and varying amounts of CSR (0–25 wt %), bonded to aluminum substrates.

The incorporation of CSR significantly increased the peel strength of the epoxy polymer composite, reaching a maximum value of 1.45 N·mm^–1^ with 5 wt % CSR, which is approximately three times higher than that of the reference (CSR0). The presence of CSR particles inhibited microcrack propagation and enhanced the overall mechanical performance of the composite.^44^ Similar improvements have been reported by Riew et al.,^45^ who added acrylic CSR (with an outer shell containing poly­(methyl methacrylate), ethyl acrylate, and acrylic acid) into epoxy resin. In their study, the peel strength of neat epoxy was 0.33 N·mm^–1^, while the maximum value achieved with methyl methacrylate–acrylonitrile-based CSR tougheners was 1.19 N·mm^–1^. Likewise, Jianwen et al.^46^ observed an increase from 0.1 N·mm^–1^ (neat epoxy) to 0.4 N·mm^–1^ with the addition of 18 wt % CSR toughening agent containing polybutadiene. Similarly, Antonino et al.^47^ reported a 3-fold improvement in T-peel strength with the incorporation of 30 wt % CSR (Kane Ace MX 153).

In the present study, T-peel strength increased with CSR content up to 10 wt %. During peeling, the CSR particles and the voids they created enhanced energy absorption and dissipation, thereby contributing to the improved fracture energy of the adhesive joint, as observed in the T-peel tests. This trend is consistent with the adhesive fracture energy values shown in Figure 7b, where the highest energy is observed at 5 and 10 wt % CSR (2803 and 2729 J/m^2^, respectively), indicating optimal toughening at these contents. However, beyond 10 wt % CSR, both peel strength and adhesive fracture energy decreased due to particle agglomeration, which likely hindered effective stress transfer. The T-peel specimen surfaces are shown in Figure 7c, revealing that the predominant failure mode was adhesive failure occurring along the substrate interface.

#### 3.1.3 Tensile Strength

Stress–strain curves of the epoxy-based polymer composites are shown in Figure 8, and the results are summarized in Table 2.

**Figure 8:**
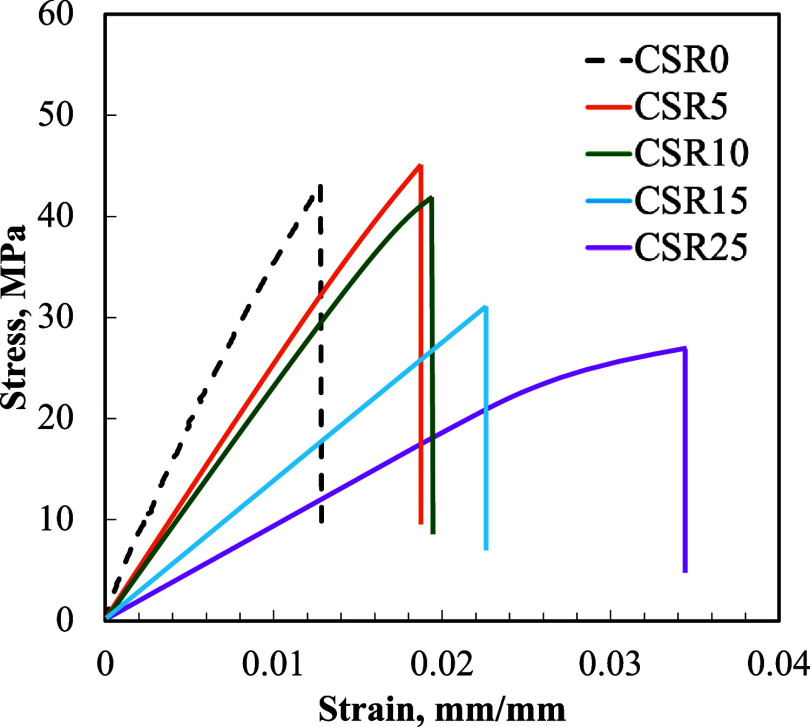
Stress–strain curves of the epoxy-based polymer composites containing 20 wt % BT and varying amounts of CSR (0–25 wt %).

**Table 2:** Tensile strength (σ_*y*_^max^), Young’s modulus (E) and Poisson’s ratio (ν) of the epoxy-based polymer composites containing 20 wt % BT and varying amounts of CSR (0–25 wt %)

compositions	σ*_y_* ^max^ (MPa)	*E* (MPa)	*ν*
CSR0 (ref)	42.6 ± 3.2	3352.2 ± 512.5	0.26 ± 0.08
CSR5	43.2 ± 5.3	2248.0 ± 569.4	0.27 ± 0.03
CSR10	39.0 ± 5.4	2155.6 ± 611.8	0.29 ± 0.13
CSR15	29.0 ± 1.9	1247.3 ± 292.0	0.31 ± 0.06
CSR25	27.6 ± 4.3	965.3 ± 247.7	0.32 ± 0.08

All samples exhibited predominantly brittle behavior, characterized by a sharp drop in stress after the maximum load and minimal plastic deformation.^48^ The observed brittle failure after incorporating CSR into the epoxy matrix was unexpected, as CSR is commonly used to enhance toughness by improving energy absorption and reducing crack propagation.^49^ While a distinct yielding was not observed in any of the samples, a progressive increase in failure strain with increasing CSR content suggests a relative improvement in ductility. This trend was also reflected by a decrease in elastic modulus, showing that the material stiffness decreased with higher CSR content.

However, the overall brittle response could be attributed to the addition of barium titanate (BT), a ceramic material, which introduces multiple interfaces within the polymer matrix. These interfaces could act as stress concentrators, promoting crack initiation and propagation, leading to brittle fracture.^50^ The combination of CSR and BT in the matrix may not be fully compatible, contributing to poor dispersion and facilitating the formation of these critical interfaces.

The tensile strength of the reference material, CSR0, was measured to be 42.6 MPa. Tensile strength was dependent on the curing agent and additives. It is also extremely sensitive to surface defects. Such imperfections likely caused the relatively low values to be measured.^16^ It is well-known that adding CSR particles can reduce the tensile strength of thermoset polymers due to the stress concentration effect of the particles.^16^ The lowest tensile strength measured was 27.6 MPa for the 20 wt % CSR particle included epoxy matrix. The decrease in tensile strength at higher CSR contents (≥15 wt %) might be attributed to multiple factors. First, the significant increase in viscosity due to the combined presence of CSR and BaTiO_3_ resulted in inhomogeneous dispersion of BaTiO_3_ particles, leading to agglomeration and stress concentration zones within the matrix. Agglomerations reduce the interfacial/interphase quality and the tensile properties of composites by decreasing the effective volume fraction of particles.^51^ Second, the reduced interparticle distance between CSR particles at higher concentrations might cause overlapping stress fields and promote void formation, creating localized zones of weakness.^52^

The reference (CSR0) had a tensile modulus of 3.35 ± 0.5 GPa. The modulus decreased approximately linearly with increasing CSR content to 0.96 ± 0.24 GPa when 25 wt % of CSR particles was added (see Table 2). Giannakopoulous et al.^49^ reported similar results using CSR particles to toughen the epoxy matrix. The modulus of butadiene rubber (i.e., the CSR core) was in the range of 1–10 MPa.^1^ Thus, the volumetric fraction of the epoxy/curing agent matrix per unit area decreased, and the modulus decreased as the CSR content increased due to the effect of the rubbery CSR core structure.^42^ The Poisson’s ratio increased due to the addition of the CSR, as expected.

#### 3.1.4 Fracture Properties

Figure 9a,b represents the values of the fracture energy, *G*_IC_, *J*-integral (*J*) and fracture toughness, *K*_IC_, for the CSR0 and varying amounts of CSR incorporated epoxy-based polymer composites. The results are summarized in Table 3.

**Figure 9:**
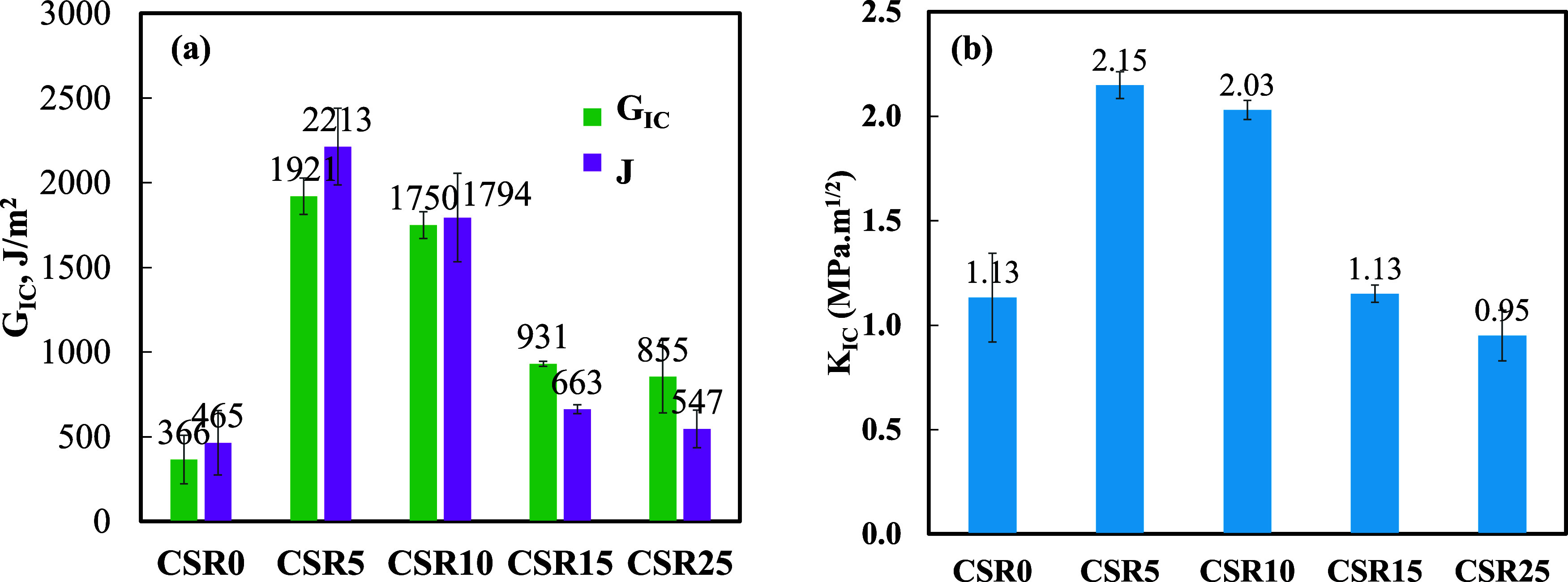
(a) Fracture energy, *J*-integral and (b) fracture toughness of the epoxy-based polymer composites containing 20 wt % BT and varying amounts of CSR (0–25 wt %).

**Table 3:** Fracture Energy, *G*_IC_, *J*-Integral, *J* and Fracture Toughness, *K*_IC_, of the Epoxy-Based Polymer Composites Containing 20 wt % BT and Varying Amounts of CSR (0–25 wt %)

compositions	*G*_IC_ (J/m^2^)	*J* (J/m^2^)	*K*_IC_ (MPa·m^1/2^)
CSR0 (ref)	366 ± 143	465 ± 190	1.13 ± 0.21
CSR5	1921 ± 108	2213 ± 226	2.15 ± 0.16
CSR10	1750 ± 79	1794 ± 261	2.03 ± 0.05
CSR15	931 ± 16	663 ± 37	1.13 ± 0.04
CSR25	855 ± 214	547 ± 111	0.95 ± 0.12

CSR0 had a mean fracture energy of 366 J/m^2^. The fracture energy increased significantly with the addition of 5 wt % CSR, reaching a maximum value of 1921 J/m^2^ due to the toughening effect provided by the rubber particles.

The addition of CSR particles increased the fracture toughness of the adhesive, as evidenced by the rise in adhesive fracture energy peaking at 5–10 wt % CSR (Figure 9a). This increase is attributed to enhanced plastic deformation around the crack tip, consistent with previous findings that the presence of rubber particles creates more zones of deformation before fracture.^53^ Gómez-del Río et al.^54^ also reported that a combination of rigid and soft particles leads to increased plastic deformation in the fracture process zone. At CSR contents above 10 wt %, the adhesive fracture energy decreased. The epoxy with CSR was procured as a commercially premixed system, which is indicative of a homogeneous distribution of CSR particles. SEM analysis confirmed this uniform dispersion. However, at higher CSR contents, the viscosity of the formulation increased significantly, making it difficult to achieve effective mixing. As a result, BT particles were not uniformly dispersed and were observed to agglomerate in the SEM images. This agglomeration corresponds with the reduction in fracture energy, highlighting the importance of proper filler dispersion for mechanical performance.

Quan et al.^17^ reported optimum fracture energy with 7 vol % CSR and 4 vol % nano additives as 2180 J/m^2^. Additionally, extensive work has investigated the effects of CSR content on the fracture properties of epoxy, and higher fracture energy values were reported.^1,16,49,52^

Based on Huang and Kinloch’s model,^54^ the reduction in fracture toughness can be explained by the increase in the size of the voids, which were cavitated rubber particles in the presence of BT in the matrix. There is an optimal limit of CSR that maximizes toughening by mechanisms such as cavitation and shear yielding. Beyond this optimal concentration, the additional CSR particles may not effectively contribute to toughening and can instead create stress concentration points.

On the other hand, incorporating CSR into the epoxy matrix may disrupt the cross-linking network of the polymer. Beyond 10 wt % CSR, an excessive amount of CSR addition may lead to a decrease in the overall cross-linking density of the epoxy matrix, which reduces the material’s ability to resist crack propagation and lowers the fracture energy.

The same trend was observed for fracture toughness values of epoxy-based polymer composites. The fracture toughness of the reference material (CSR0), calculated by eq 2, was 1.13 ± 0.21 MPa·m^1/2^, similar to the values reported in the literature for epoxy resins.^1,33,55,56^ In our study, as the number of CSR particles increased, there was an improvement in fracture toughness up to 5 wt % CSR incorporation (2.15 MPa·m^1/2^) due to the higher number of deformation zones before fracture. Micron-sized CSR particles are effective in increasing the extended shear yielding of the epoxy matrix, resulting in plastic deformation.^53^ There is general agreement that the increase in toughness is due to the plastic growth of the voids, which results from the failure of the CSR particles and the progress of shear bands between these voids.^9^ However, in this study, beyond 10 wt % CSR addition, fracture toughness decreased due to a decrease in cross-link density of the epoxy matrix, agglomerations and a possible change in the morphology.

### 3.2 Dynamic Mechanical Thermal Analyses

Table 4 shows the DMTA results of the bulk epoxy-based polymer composites as a function of CSR content in the tension mode. *T*_g_ values (obtained from the peak of tan δ) increased upon the incorporation of CSR particles. In the present study, all formulations include 20 wt % BaTiO_3_, and the reference material (CSR0), which contains no CSR, represents the “only BT” system. Therefore, the comparison between CSR0 and other formulations highlights the additional effect of CSR on *T*_g_.

**Table 4:** Glass Transition Temperature (*T*_g_), Storage Modulus (*E*’), Crosslink Density (*ν_XL_*) of the Epoxy-Based Polymer Composites Containing 20 wt % BT and Varying Amounts of CSR (0–25 wt %)

compositions	*T*_g_ (tan δ), °C	*E*’, MPa	*ν_XL_*, mmol/cm^3^	constrained region (C)
CSR0	126.1 ± 0.5	2880.5 ± 317.2	709.6 ± 86.7	
CSR5	140.9 ± 0.3	2580.1 ± 104.2	1650.8 ± 73.1	0.104
CSR10	141.0 ± 5.4	2380.5 ± 278.1	1304.1 ± 505.8	0.079
CSR15	141.3 ± 3.5	1794.3 ± 272.3	1364.6 ± 128.1	0.108
CSR25	142.8 ± 2.9	1198.3 ± 70.73	1105.2 ± 98.53	0.118

The *T*_g_ of CSR0 (reference) was 126.1 ± 0.5 °C and increased to 140.9 ± 0.3 °C with the addition of 5 wt % CSR, representing a notable shift (Figure 10a). This initial increase is attributed to improved damping behavior and energy dissipation capability introduced by the CSR, in agreement with the literature.^42^ However, further additions of CSR (CSR10–CSR25) resulted in only minor changes in *T*_g_ (remaining within almost 2 °C).

**Figure 10:**
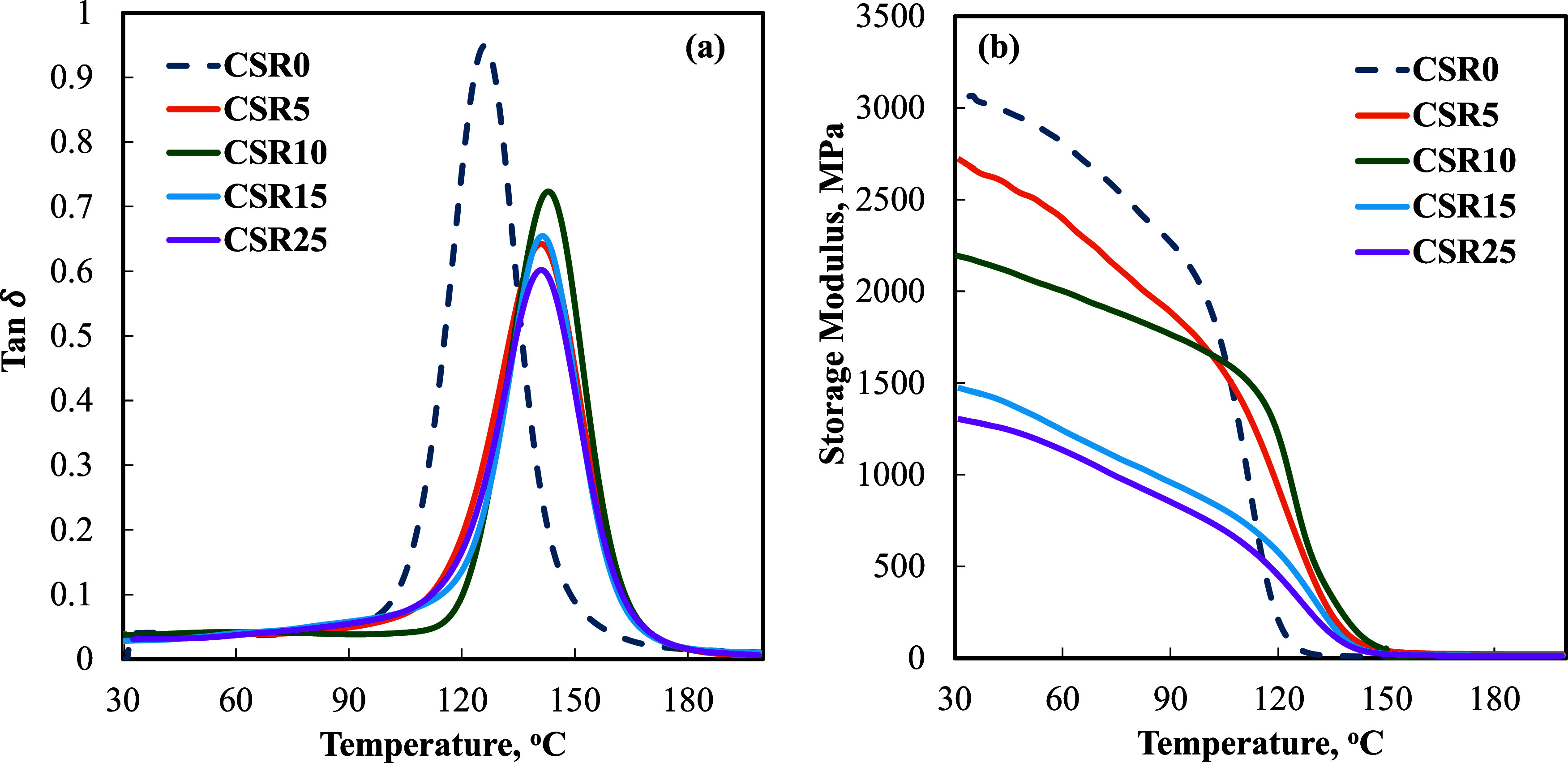
*T*_g_ (tan δ) (a) and storage modulus (b) of the epoxy-based polymer composites containing 20 wt % BT and varying amounts of CSR (0–25 wt %).

Quan and Ivankovic^1^ obtained similar results with the reference material’s *T*_g_ as 122.5 °C, and they observed an increase in *T*_g_ with the addition of CSR due to the strong interaction between epoxy and CSR. However, He et al.^57^ investigated the *T*_g_ of rubber-modified epoxy resin and noted minimal variation in *T*_g_ across the entire range of rubber concentrations (from 0 to 67 vol %). This stability in *T*_g_ was attributed to the fact that the rubber phase remained separated and did not significantly interfere with the segmental dynamics or cross-linking density of the epoxy matrix. Giannakopoulos et al.^49^ demonstrated that the addition of 9 wt % of CSR had no significant impact on *T*_g_, while Chen et al.^16^ reached a similar conclusion, reporting that the inclusion of polysiloxane-based core–shell particles up to 25 vol % had no notable effect on the epoxy’s *T*_g_.

A similar ternary system composed of CSR and silica nanoparticles was studied by Quan et al.,^17^ where a comparable increase in *T*_g_ was observed due to CSR addition, while silica nanoparticles had a negligible impact. Rigid fillers can restrict polymer chain mobility through interfacial interactions. However, as suggested in their study,^17^ the *T*_g_ increase in such ternary systems is typically attributed primarily to the rubber–matrix interaction rather than the ceramic phase. Therefore, the more significant *T*_g_ increase observed here at 5 wt % CSR was mainly due to the influence of CSR.

A large reduction in the height of the tan δ peak was observed in Figure 10a, compared to CSR0. This behavior is frequently associated with increased constrained chain regions and reduced molecular mobility, as commonly reported in rubber-modified epoxy systems.^58^ Furthermore, the broadening of the tan δ peak observed at higher CSR content suggests a broader relaxation time distribution and a more heterogeneous network structure, as also reported by Quan and Ivankovic.^59^

To quantitatively assess the immobilized polymer fraction, the constrained region (C) was calculated based on the peak height of the tan δ curve using the method described by Poornima Vijayan et al.^39^ In this analysis, the epoxy composite containing 20 wt % BaTiO_3_ and no CSR (CSR0) was taken as the reference, assuming it exhibits a fully amorphous response (C=0). Although BaTiO_3_ itself contributes to a degree of physical restriction due to its inorganic surface, this baseline system was used consistently for relative comparison. As the CSR content increased from 5 to 25 wt %, a progressive decrease in tan δ peak height and energy loss fraction was observed, indicating a reduction in segmental chain mobility. This behavior is attributed to the formation of interfacial constrained regions around the dispersed CSR particles, which physically restrict polymer relaxation during the glass transition. Accordingly, the calculated C values increased from 0.10 to 0.12, confirming the growing immobilized fraction despite a decrease in cross-link density and storage modulus.

The storage modulus decreased as the CSR content increased (Figure 10b) since the existence of soft rubbery particles reduces the stiffness of the relatively rigid polymer.^15,16,49,60,61^ The maximum storage modulus value was obtained with the reference material at 2880.5 MPa, and it decreased to 1198.3 MPa with the addition of 25 wt % CSR.

The cross-link density of the reference material was calculated as 709 mmol/cm^3^ and increased significantly by 5 wt % CSR incorporation (1650.8  ±  73.1 mmol/cm^3^). Beyond this point, a gradual decrease in cross-link density was observed at higher CSR contents. It should be noted that the Flory theory was originally developed for homogeneous polymer networks and does not account for the presence of rigid fillers or rubber modifiers. As such, the calculated values represent an apparent cross-link density, influenced not only by the chemical network structure but also by physical effects such as particle–matrix interactions and changes in the overall stiffness of the polymer composite. The CSR particles used are designed for epoxy systems, and related patent literature^62^ indicates that the shell phase may contain functional groups capable of reacting with epoxy or hardener components. The initial increase in cross-link density may have contributed to the improved mechanical performance at 5 wt % CSR, whereas the subsequent decrease may explain the reduction in fracture toughness and fracture energy observed at higher loadings. This trend is consistent with the findings of Liu et al.,^63^ who showed that higher cross-link density enhances the toughness of epoxy systems by enabling more efficient energy dissipation. Similarly, as Quan et al.^17^ reported, further addition of CSR beyond an optimal content did not improve the properties further, which was attributed to saturation of the toughening mechanism and reduced efficiency of interfacial interactions. A similar mechanism may explain the decrease in cross-link density and the corresponding reduction in fracture energy at higher CSR levels in the present study.

### 3.3 Toughening Mechanisms

Figure 11 shows the fracture surfaces of the epoxy-based polymer composites for the 3PB fracture toughness specimens. The reference material’s surface (CSR0 – Figure 11a) was found to be smooth and glassy, typical behavior of brittle thermoset polymers.^64^ Low fracture energy values were obtained for this material since no large-scale plastic deformation occurred during fracture. Cracks on the surface were observed due to the excess energy associated with the relatively fast crack growth. In this case, subsurface crack formation absorbs the excess energy during the fracture of brittle materials.^16^ Although a relatively homogeneous distribution of BT was obtained, some BT agglomerates exist (shown in Figure 11a) due to incomplete dispersion, which affected the mechanical response of the epoxy as represented in Tables 2 and 3. This observation was further quantified in Table S1, where image-based BT particle analysis (Figures S1–S5) confirmed an increasing degree of agglomeration with increasing CSR content, reaching 3.58% BT area in CSR25 compared to 0.5% in CSR0. This behavior was attributed to the increased viscosity introduced by CSR, which hindered the efficient dispersion of BT particles during mixing and promoted their local accumulation. Thus, despite a constant BT loading across all formulations, CSR-rich systems exhibited poorer BT dispersion due to limited mobility during mixing.

**Figure 11:**
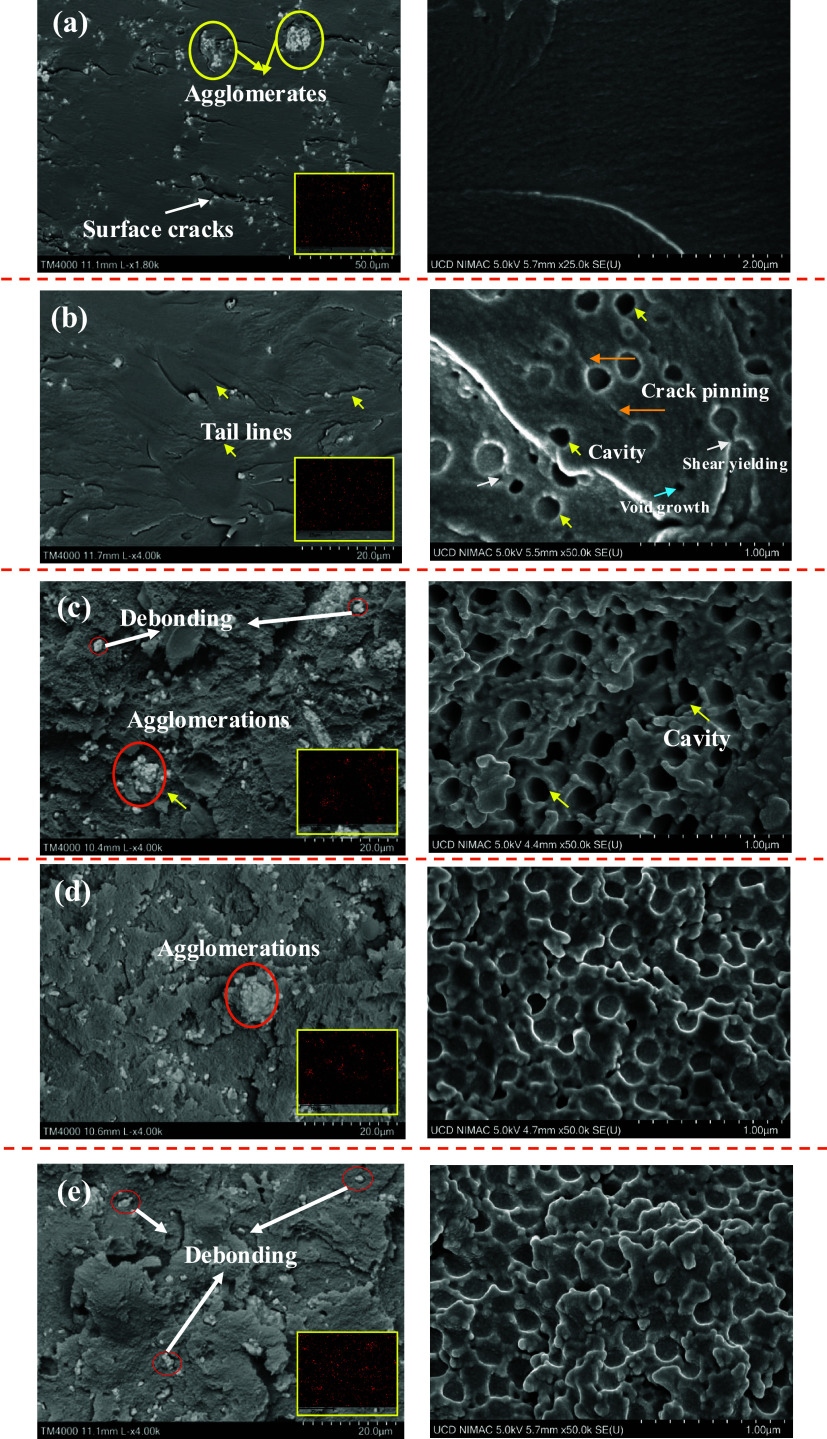
SEM micrographs of fracture surfaces of epoxy-based polymer composites containing 20 wt % BT and varying amounts of CSR: (a) CSR0 (with the image of BT distributions – orange dots), (b) CSR5, (c) CSR10, (d) CSR15, (e) CSR20 (with the image of BT distributions – red dots). Scale bars and magnifications: (a) 50 μm/×1800 and 2 μm/×25,000; (b–e) 20 μm/×4000 and 1 μm/×50,000.

As the CSR content increased, the fracture surface exhibited a more porous and rougher morphology. The cavities caused by CSR were analyzed using ImageJ, revealing a measured diameter of 240 ± 81 nm. The cavity size did not show a direct correlation with BT content but instead with CSR content. Examination of the fracture surface showed that the matrix displayed enhanced ductility, allowing for more effective deformation transfer between the matrix and CSR particles. This behavior led to the yielding of CSR particles under stress.^42^ Detailed morphological metrics were measured through image analysis to quantify the CSR area percentage within the matrix, as presented in Table S2 and Figures S6–S9. As the CSR content increased, the CSR area within the structure increased proportionally.

The distribution of CSR particles influences the observed toughening mechanisms. In regions where CSR particles were sparsely distributed, isolated cavitation zones were identified (Figure 11a). These large interparticle distances limit the interaction between cavitation-induced plastic zones, potentially reducing overall energy dissipation. This observation is consistent with the findings of Giannakopoulos et al.,^49^ who reported that closer spacing between CSR particles facilitates cooperative plastic deformation, thereby enhancing fracture toughness. In the present study, CSR5 exhibited the highest fracture energy, indicating that toughening mechanisms such as plastic deformation were most effective at this composition. However, at higher CSR contents, a decrease in fracture energy was observed despite the rougher fracture surface and the closer interparticle distances. This could be attributed to the agglomeration of BT particles. Such agglomerations create stress-concentrated areas within the polymer matrix, which serve as sites of structural weakness and promote crack initiation.^65^ The distribution of BT particles was observed as represented on the bottom right side of Figures 11 and S1–S5.

Additionally, the observation of tail lines (Figure 11b) around micron-sized BT in CSR5 samples indicates a typical failure plane of micron-sized particles within the epoxy matrix. This occurs as cracks propagate through the matrix and deflect around the BT particles, following the path of least resistance. Quan et al.^17^ explained that these deflections, which are indicated by the tail lines, significantly increase the surface area of the fracture surface. These tail lines, which appear as elongated streaks extending from particles, resemble a comet’s tail and indicate the direction of crack propagation.^66^ They occur between the matrix and the particles, and they show how cracks navigate through or around the particles during fracture. To visualize the toughening mechanisms, a schematic representation is provided in Figure 12, mapping the interactions of CSR cavitation, crack pinning, shear yielding, BT-induced deflection, and crack propagation features (tail lines and debonding zones).

**Figure 12:**
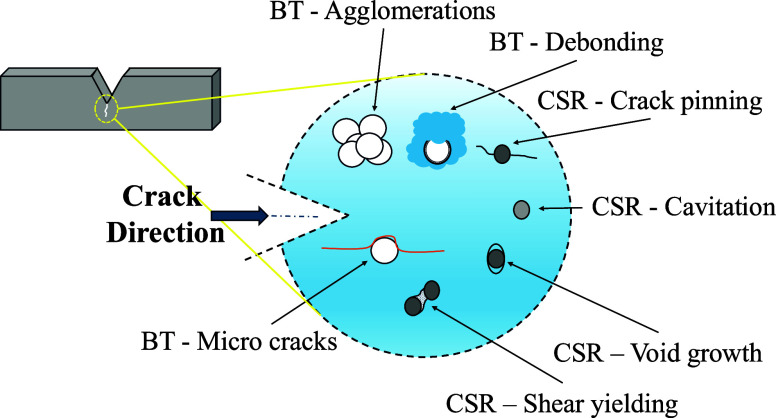
Different toughening mechanisms observed near the crack tip in CSR and BT incorporated epoxy systems under three-point bending (3PB) testing.

Tail lines play a crucial role in indicating surface tortuosity and crack twisting; when the angle of twist reaches its maximum, the crack front propagates at different elevations. This creates additional surface area.^56^ Kitey and Tippur^66^ explained in their study that for strongly bonded particles, tail lines are more frequent in the matrix between particles. In contrast, for weakly bonded particles, tail lines appear at the particles due to the weaker filler–matrix interface acting as a crack attractor. These features lead to an increase in surface roughness and energy dissipation during fracture. The effects are more pronounced in samples containing weakly bonded particles, owing to the higher occurrence of crack twisting. The deflection caused by tail lines was not observed for CSR10 and the higher amount of CSR-containing composites. The area of the composites’ failure plane was equal to the epoxy matrix. Unlike CSR5 samples, voids generated by CSR debonding or cavitation are clearly seen after 10 wt % CSR addition. Therefore, tensile strength decreased. Quan et al.^17^ referred to the voids generated by CSR as a result of rubber cavitation. As the rubber content increased, the distance between rubber additives decreased, leading to increased interaction between the rubber particles and BT. Consequently, the fracture toughness increased up to a certain level of CSR addition. Notably, despite the reduction in fracture toughness beyond 5 wt % CSR, all modified formulations exhibited higher values than CSR0, including the formulation with the highest CSR content.

### 3.4 X-ray Diffraction and Voltage Output Test

BT is a well-known dielectric material with five different crystal systems. Among them, the tetragonal asymmetrical structure shows an excellent piezoelectric effect.^67^ When it is incorporated into the composite, BT enhances the dielectric constant of the material. Joshi et al.^68^ reported that BT shows a phase transition as a function of temperature. Thus, maintaining the tetragonal phase of BT throughout the process is crucial. Figure 13a shows the X-ray diffraction analysis of the reference material (CSR0) and confirms that the tetragonal phase remained unchanged at the processing temperature. The tetragonal crystalline phase of BT was confirmed by characteristic XRD peaks at 31° (110 tetragonal planes), 22° (100), splitting at 45° (200 tetragonal planes), and 50.8° (210 tetragonal planes), indicating that the 100 °C process temperature did not lead to phase transformation, and the piezoelectric tetragonal phase remained in the structure.

**Figure 13:**
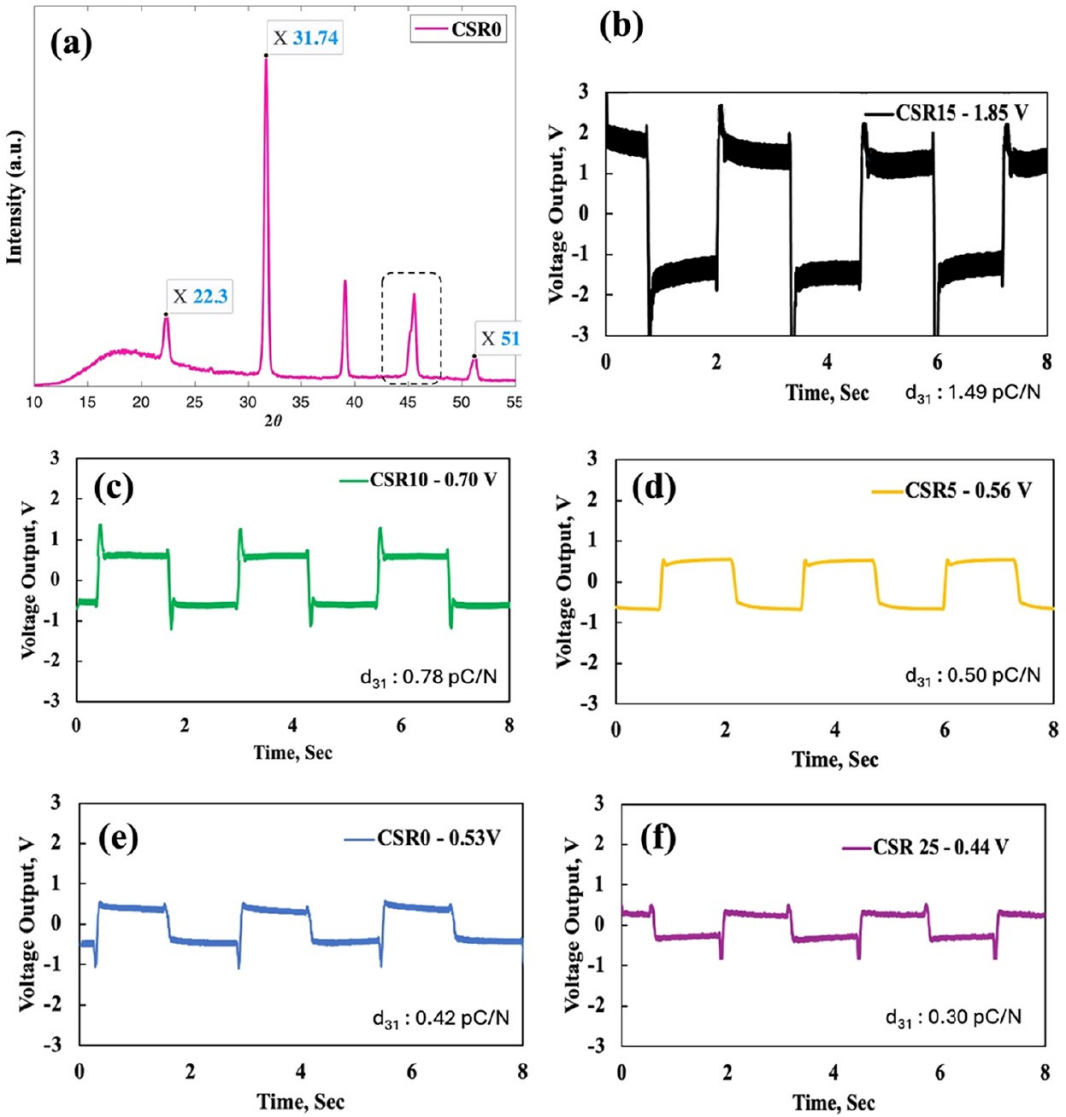
(a) XRD of CSR5 with tetragonal crystalline structure of BT; Voltage output test results and d_31_ values: (b) CSR15, (c) CSR10, (d) CSR5, (e) CSR0, (f) CSR25.

The voltage output test results are given in Figure 13. The incorporation of 15 wt % CSR resulted in increased ductility, as evidenced by higher elongation at break. Microscopic analysis revealed the presence of barium titanate agglomerates within the epoxy matrix, indicating nonuniform particle distribution. As noted above, these agglomerates were consistently observed throughout the composite, suggesting that the CSR phase did not effectively facilitate the dispersion of BT particles. Despite this, an increased piezoelectric response was measured, coinciding with the presence of larger BT clusters. A maximum of 1.85 V was achieved with CSR15 epoxy-based polymer composites.

Kargarzadeh et al.^53^ studied flexible piezoelectric sensors using BT-CNT/silicone rubber combinations. They poled the films at 140 °C for 30 min under an electric field of 12 kV/mm, and a maximum output voltage of 4 V was obtained under a 10 N applied force. They identified the optimal BT incorporation as 20 wt %, beyond which the results showed a decrease in voltage generation due to an increase in Young’s modulus. The aggregation was found to decrease the sensitivity of the film. Wang et al.^69^ developed sandwich structure nanogenerators incorporating BT, CNT, and β phase polarized PVDF. Their investigation in bending mode yielded a 9.8 V output using BT alone. Similarly, Zhang et al.^70^ prepared a piezoelectric paper based on BT nanoparticles and bacterial cellulose, reporting an open circuit voltage of 14 V. Under cyclic bending conditions, the piezoelectric material generated a peak voltage of 1.5 V. Kim et al.^71^ investigated high-performance flexible piezoelectric pressure sensors based on 0–3 type CNT-doped PZT/epoxy composites. Their study on the effect of CNT content on the piezoelectric response revealed a peak output of 1.5 V under compressive loading at 0.07 wt % CNT and 80 wt % PZT. On the other hand, Vittayakorn et al.^72^ developed BaTiO_3_/epoxy resin nanocomposites for use as flexible energy storage devices and reported a relatively high open-circuit voltage of approximately 105 V for a 60 wt % BT/epoxy sample under 150 N mechanical loading at 2.5 Hz. This high performance was primarily attributed to the large dielectric constant of BaTiO_3_. However, the authors also noted a reduction in breakdown strength at higher filler contents, likely due to nanoparticle agglomeration and increased porosity. Notably, the study did not mention any poling treatment of the samples, and the BaTiO_3_ used was in the cubic phase, which is generally nonpiezoelectric.

In this study, CSR particles may induce interfacial polarization due to the difference in permittivity between the rubber phase and the surrounding matrix. At higher concentrations of CSR, BT particles agglomerate, creating discontinuities in the piezoelectric charge pathways and hindering the movement of charge carriers, thereby reducing the voltage output. Furthermore, excessive amounts of CSR may lead to increased dielectric loss in the composite, which could diminish the material’s efficiency in converting mechanical energy into electrical energy, resulting in lower voltage output.

## 4 Conclusion

The mechanical, fracture, thermal and voltage output properties of the epoxy composite with fixed barium titanate and a varying amount of core–shell rubber were studied. The addition of CSR decreased Young’s modulus and tensile strength while increasing the glass transition temperature.

The fracture behavior of the polymer composite was investigated by a three-point bending test. Fracture energy of 366 J/m^2^ was measured for the reference material, CSR0. The incorporation of CSR improved the fracture energy of the composites, with the highest value obtained at 5 wt % CSR loading. Scanning electron microscopy of the fracture surfaces showed that core-to-shell debonding was followed by plastic void growth of the epoxy.

XRD analysis confirmed that the tetragonal crystalline structure of barium titanate was preserved after composite processing. This was attributed to the controlled processing temperature (maintained at 100 °C), which prevented phase transformation and degradation of the piezoelectric properties of BT. Piezoelectric voltage output and d_31_ analyses were performed to evaluate the piezoelectric properties of the composites. The results demonstrated that adding CSR and BT particles enhanced the piezoelectric performance of the composites, with the highest voltage output and d_31_ values obtained at 15 wt % CSR loading. The addition of CSR and BT additives in epoxy composites led to enhanced fracture toughness and piezoelectric performance. Overall, the optimized formulation containing 15 wt % CSR provided an effective balance between mechanical toughness and piezoelectric responsiveness. This formulation is particularly promising for use in applications such as flexible piezoelectric sensors, structural health monitoring systems, and wearable electronics, where both mechanical durability and signal sensitivity are essential.

## Supplementary Material



## Data Availability

The data underlying this study are not publicly available due to commercial confidentiality. However, the data that support the findings of this study are available from the corresponding author upon request.

## Notes

% Area of barium titanate as a function of CSR content (Table S1); % Area of core–shell rubber as a function of CSR content (Table S2); BT dispersion analysis using ImageJ at varying CSR contents (Figures S1–S5); CSR dispersion analysis using ImageJ at varying CSR contents (Figures S6–S9) (PDF)

## ∥

Luxembourg Institute of Science and Technology, Hautcharage, L-4940, Luxembourg

## References

[1] Quan D., Ivankovic A. (2015). Effect of core–shell rubber (CSR) nano-particles
on mechanical properties and fracture toughness of an epoxy polymer. Polymer.

[2] Bajpai A., Alapati A., Klingler A., Wetzel B. (2018). Tensile Properties,
Fracture Mechanics Properties and Toughening Mechanisms of Epoxy Systems
Modified with Soft Block Copolymers, Rigid TiO2 Nanoparticles and
Their Hybrids. J. Compos. Sci..

[3] Abadyan M., Khademi V., Bagheri R., Haddadpour H., Kouchakzadeh M. A., Farsadi M. (2009). Use of rubber modification
technique
to improve fracture-resistance of hoop wound composites. Mater. Des..

[4] Marouf B. T., Pearson R. A., Bagheri R. (2009). Anomalous
fracture behavior in an
epoxy-based hybrid composite. Mater. Sci. Eng.:
A.

[5] Zhou H., Xu S. (2014). A new method to prepare rubber toughened
epoxy with high modulus
and high impact strength. Mater. Lett..

[6] Ratna D., Banthia A. K. (2004). Rubber toughened
epoxy. Macromol.
Res..

[7] Kinloch A. J., Yuen M. L., Jenkins S. D. (1994). Thermoplastic-toughened epoxy polymers. J. Mater. Sci..

[8] Deng S., Ye L., Friedrich K. (2007). Fracture behaviours
of epoxy nanocomposites with nano-silica
at low and elevated temperatures. J. Mater.
Sci..

[9] Mousavi S. R., Estaji S., Raouf Javidi M., Paydayesh A., Khonakdar H. A. (2021). Toughening of epoxy
resin systems using core–shell
rubber particles: a literature review. J. Mater.
Sci..

[10] Wang F., Drzal L. T., Qin Y., Huang Z. (2016). Enhancement of fracture
toughness, mechanical and thermal properties of rubber/epoxy composites
by incorporation of graphene nanoplatelets. Composites, Part A.

[11] Dou H., Tian B., Huang Y., Quan Y., Chen Q., Yin G. (2016). Improved mechanical
properties of ATBN-toughened epoxy networks by
controlling the phase separation scale. J. Adhes.
Sci. Technol..

[12] Fakhar A., Seyed Salehi M., Keivani M., Abadyan M. (2016). Comprehensive
Study
on Using VTBN Reactive Oligomer for Rubber Toughening of Epoxy Resin
and Composite. Polym.-Plast. Technol. Eng..

[13] Bagheri R., Marouf B. T., Pearson R. A. (2009). Rubber-Toughened Epoxies: A Critical
Review. Polym. Rev..

[14] Bajpai A., Wetzel B., Friedrich K. (2020). High strength
epoxy system modified
with soft block copolymer and stiff core-shell rubber nanoparticles:
Morphology, mechanical properties, and fracture mechanisms. Express Polym. Lett..

[15] Becu L., Maazouz A., Sautereau H., Gerard J. F. (1997). Fracture behavior
of epoxy polymers modified with core-shell rubber particles. J. Appl. Polym. Sci..

[16] Chen J., Kinloch A. J., Sprenger S., Taylor A. C. (2013). The mechanical properties
and toughening mechanisms of an epoxy polymer modified with polysiloxane-based
core-shell particles. Polymer.

[17] Quan D., Pearson R. A., Ivankovic A. (2018). Interaction of toughening mechanisms
in ternary nanocomposites. Polym. Compos..

[18] Jo E.-B., Lee Y.-A., Cho Y.-A., Günther P. A. (2023). The 0–3 Lead Zirconate-Titanate
(PZT)/Polyvinyl-Butyral (PVB)
Composite for Tactile Sensing. Sensors.

[19] Xie S.-H., Zhu B.-K., Wei X.-Z., Xu Z.-K., Xu Y.-Y. (2005). Polyimide/BaTiO3
composites with controllable dielectric properties. Composites, Part A.

[20] Yasar M., Hassett P., Murphy N., Ivankovic A. (2024). β Phase
Optimization of Solvent Cast PVDF as a Function of the Processing
Method and Additive Content. ACS Omega.

[21] Yasar M., Sanay B., Duffy C., Thai M., Murphy N. (2025). A study of epoxy based
polymer composites: Tailoring mechanical and
piezoelectric characteristics. Polym. Compos..

[22] Yasar M., Murphy N., Ivankovic A. (2023). Development and Characterization
of Epoxy-Based Polymer Composites Containing Piezoelectric and Electrically
Conductive Fillers. IEEE Int. Symp. Appl. Ferroelectr..

[23] Xu Y., Chung D. D. L., Mroz C. (2001). Thermally
conducting aluminum nitride
polymer-matrix composites. Composites, Part
A.

[24] Zhang X., Le M.-Q., Zahhaf O., Capsal J.-F., Cottinet P.-J., Petit L. (2020). Enhancing dielectric and piezoelectric
properties of micro-ZnO/PDMS
composite-based dielectrophoresis. Mater. Des..

[25] Manchi P., Graham S. A., Patnam H., Alluri N. R., Kim S.-J., Yu J. S. (2021). LiTaO_3_-Based Flexible Piezoelectric Nanogenerators for
Mechanical Energy Harvesting. ACS Appl. Mater.
Interfaces.

[26] Lemaire E., Thuau D., De Vaulx J.-B., Vaissiere N., Atilla A. (2021). Rochelle Salt-Based Ferroelectric and Piezoelectric
Composite Produced with Simple Additive Manufacturing Techniques. Materials.

[27] Maji S., Asrey R., Kumar S., Saxena C., Kumar N. (2010). Polymer-coated piezoelectric quartz crystal sensor for sensing the
nerve agent simulant dimethyl methylphosphonate vapor. J. Appl. Polym. Sci..

[28] Meisak D., Kinka M., Plyushch A., Macutkevič J., Zarkov A., Schaefer S. (2023). Piezoelectric
Nanogenerators
Based on BaTiO _3_ /PDMS Composites for High-Frequency Applications. ACS Omega.

[29] Nayak S., Chaki T. K., Khastgir D. (2014). Development of Flexible
Piezoelectric
Poly­(dimethylsiloxane)–BaTiO _3_ Nanocomposites for
Electrical Energy Harvesting. Ind. Eng. Chem.
Res..

[30] Chen C.-H., Jian J.-Y., Yen F.-S. (2021). Morphology, thermal, and mechanical
properties of κ-aluminum oxide/CTBN/epoxy nanocomposites. Polym. Bull..

[31] Ma H., Geng P., Xu T., Kumar Bandaru A., Aravand A., Falzon B. G. (2024). Analytical fracture
toughness model
for multiphase epoxy matrices modified by thermoplastic and carbon
nanotube/thermoplastic. Composites, Part A.

[32] Zhu Z., Chen H., Chen Q., Liu C., Noh K. (2022). Fracture behavior of hybrid epoxy nanocomposites
based on multi-walled
carbon nanotube and core-shell rubber. Nano
Mater. Sci..

[33] Bajpai A., Martin R., Faria H., Ibarboure E., Carlotti S. (2021). Epoxy based hybrid nanocomposites:
Fracture mechanisms,
tensile properties and electrical properties. Mater. Today: Proc..

[34] Di C., Yu J., Wang B., Lau A. K. T., Zhu B., Qiao K. (2019). Study of Hybrid
Nanoparticles Modified Epoxy Resin Used in Filament Winding Composite. Materials.

[35] Source for IC Peel Mechanical Engineering Department, Imperial College: London; 2006 https://www.imperial.ac.uk/mechanical-engineering/research/mechanics-of-materials/composites-adhesives-and-soft-solids/adhesion/test-protocols.

[36] Kinloch A. J., Lau C. C., Williams J. G. (1994). The peeling
of flexible laminates. Int. J. Fract..

[37] ASTM . Standard test methods for plane-strain fracture toughness and strain energy release rate of plastic materials ASTM D5045–99 2007.

[38] Flory P. J. (1942). Thermodynamics
of High Polymer Solutions. J. Chem. Phys..

[39] Poornima Vijayan, P. ; Puglia, D. ; Kenny, J. ; Thomas, S. Effect of organically modified nanoclay on the miscibility, rheology, morphology and physical properties of diglycidyl ether of bisphenol-A epoxy/carboxyl-terminated (butadiene-co-acrylonitrile) blend Soft Matter 2013.

[40] Sirohi J., Chopra I. (2000). Fundamental Understanding
of Piezoelectric Strain Sensors. J. Intell.
Mater. Syst. Struct..

[41] Rangaswamy H., Sogalad I., Basavarajappa S., Acharya S., Manjunath
Patel G. C. (2020). Experimental analysis and prediction of strength of
adhesive-bonded single-lap composite joints: Taguchi and artificial
neural network approaches. SN Appl. Sci..

[42] Baek D., Sim K.-B., Kim H.-J. (2021). Mechanical
Characterization of Core-Shell
Rubber/Epoxy Polymers for Automotive Structural Adhesives as a Function
of Operating Temperature. Polymers.

[43] Georgiou, I. ; Ivankovic, A. ; Kinloch, A. J. ; Tropsa, V. Rate Dependent Fracture Behaviour of Adhesively Bonded Joints. In European Structural Integrity Society; Blackman, B. R. K. ; Pavan, A. ; Williams, J. G. , Eds.; Elsevier, 2003; Vol. 32, pp 317–328 10.1016/S1566-1369(03)80105-X.

[44] Panthakkal
Abdul Muthalif M., Choe Y. (2022). Adhesive and Impact-Peel Strength
Improvement of Epoxy Resins Modified with Mono and Diamine Functionalized
Elastomers. Adv. Polym. Technol..

[45] Riew, C. K. ; Siebert, A. R. ; Smith, R. W. ; Fernando, M. ; Kinloch, A. J. Toughened Epoxy Resins: Preformed Particles as Tougheners for Adhesives and Matrices. In Advances in Chemistry; Toughened Plastics II, American Chemical Society, 1996; Vol. 252, pp 33–44 10.1021/ba-1996-0252.ch003.

[46] Jianwen Z., Yizhou H., Hong W., Yi H. (2019). Practical Technology
of Toughening Epoxy Resin: Influence of Toughening Agents on Mechanical
and Heat Properties. Int. J. Ind. Manuf. Syst.
Eng..

[47] Antonino L. D., Garcia G. E. S., de
Oliveira Viani C., Gouveia J. R., Vidotti S. E., dos Santos D. J. (2021). Effects
of core–shell and reactive liquid rubbers
incorporation on practical adhesion and fracture energy of epoxy adhesives. Iran Polym. J..

[48] Mouritz, A. P. 18 - Fracture processes of aerospace materials. In Introduction to Aerospace Materials; Woodhead Publishing, 2012; pp 428–453 10.1533/9780857095152.428.

[49] Giannakopoulos G., Masania K., Taylor A. C. (2011). Toughening of epoxy using core–shell
particles. J. Mater. Sci..

[50] Roy A., Panda S., Gupta J., Anu, Singh R. P. (2023). Effects
of interfacial interactions on structural, optical, thermal degradation
properties and photocatalytic activity of low-density polyethylene/BaTiO3
nanocomposite. Polymer.

[51] Zare Y., Rhee K. Y., Hui D. (2017). Influences of nanoparticles
aggregation/agglomeration
on the interfacial/interphase and tensile properties of nanocomposites. Composites, Part B.

[52] Bagheri R., Pearson R. A. (1996). Role of particle
cavitation in rubber-toughened epoxies:
1. Microvoid toughening. Polymer.

[53] Kargarzadeh, H. ; Ahmad, I. ; Abdullah, I. Mechanical Properties of Epoxy/Rubber Blends. In Handbook of Epoxy Blends; Parameswaranpillai, J. ; Hameed, N. ; Pionteck, J. ; Woo, E. M. , Eds.; Springer International Publishing: Cham, 2017; pp 279–314 10.1007/978-3-319-40043-3_11.

[54] Gómez-del
Río T., Salazar A., Pearson R. A., Rodríguez J. (2016). Fracture behaviour
of epoxy nanocomposites modified with triblock copolymers and carbon
nanotubes. Composites, Part B.

[55] Tang L.-C., Zhang H., Sprenger S., Ye L., Zhang Z. (2012). Fracture mechanisms
of epoxy-based ternary composites filled with rigid-soft particles. Compos. Sci. Technol..

[56] Glaskova-Kuzmina T., Stankevics L., Tarasovs S., Sevcenko J., Špaček V. (2022). Effect of Core–Shell Rubber Nanoparticles on the Mechanical
Properties of Epoxy and Epoxy-Based CFRP. Materials.

[57] He J., Raghavan D., Hoffman D., Hunston D. (1999). The influence of elastomer
concentration on toughness in dispersions containing preformed acrylic
elastomeric particles in an epoxy matrix. Polymer.

[58] Konnola R., Parameswaranpillai J., Joseph K. (2016). Mechanical, thermal, and viscoelastic
response of novel in situ CTBN/POSS/epoxy hybrid composite system. Polym. Compos..

[59] Quan D., Ivankovic A. (2019). The curing
behaviour and thermo-mechanical properties
of core–shell rubber-modified epoxy nanocomposites. Polym. Polym. Compos..

[60] Yasar M., Celebi H., Bayram G. (2022). Role of formulation
additives on
the properties of thermoplastic polyether ester elastomer-based and
carbon fabric-reinforced multilayer composites. J. Thermoplast. Compos. Mater..

[61] Yasar, M. ; Bayram, G. ; Celebi, H. Effect of Carbon Black And/or Elastomer on Thermoplastic Elastomer-based Blends and Composites, Proceedings of PPS-30: The 30th International Conference of the Polymer Processing Society – Conference Papers, Cleveland, Ohio, USA, 2015; p 120003 10.1063/1.4918493.

[62] Lutz, A. ; Steiner, B. Accelerated and toughened two part epoxy adhesives. WO2014035655A2, 2014 https://patents.google.com/patent/WO2014035655A2/en (accessed Jun 19, 2025).

[63] Liu J. D., Sue H.-J., Thompson Z. J., Bates F. S., Dettloff M. (2009). Effect of crosslink
density on fracture behavior of model epoxies
containing block copolymer nanoparticles. Polymer.

[64] Hsieh T. H., Kinloch A. J., Masania K., Taylor A. C., Sprenger S. (2010). The mechanisms
and mechanics of the toughening of epoxy polymers modified with silica
nanoparticles. Polymer.

[65] Gupta
K B. N. V. S. G., Patnaik S., Prusty R. K., Ray B. C. (2023). Simultaneous
enhancement in interlaminar– shear strength and fracture toughness
through nano Al2O3 dispersion in glass fiber/IPN multiscale composites. Composites, Part A.

[66] Kitey R., Tippur H. V. (2005). Role of particle
size and filler–matrix adhesion
on dynamic fracture of glass-filled epoxy. II. Linkage between macro-
and micro-measurements. Acta Mater..

[67] Zheng Y., Zhao L., Li Y., Zhang X., Zhang W., Liu L. (2023). Nanostructure Mediated Piezoelectric Effect of Tetragonal
BaTiO3 Coatings on Bone Mesenchymal Stem Cell Shape and Osteogenic
Differentiation. Int. J. Mol. Sci..

[68] Joshi U. A., Yoon S., Baik S., Lee J. S. (2006). Surfactant-Free
Hydrothermal Synthesis of Highly Tetragonal Barium Titanate Nanowires:
A Structural Investigation. J. Phys. Chem. B.

[69] Wang Y., Zhang X., Guo X., Li D., Cui B., Wu K. (2018). Hybrid nanogenerator
of BaTiO3 nanowires and CNTs for
harvesting energy. J. Mater. Sci..

[70] Zhang G., Liao Q., Zhang Z., Liang Q., Zhao Y. (2016). Novel Piezoelectric
Paper-Based Flexible Nanogenerators Composed
of BaTiO3 Nanoparticles and Bacterial Cellulose. Adv. Sci..

[71] Kim H. J., Kim Y. J. (2018). High performance
flexible piezoelectric pressure sensor
based on CNTs-doped 0–3 ceramic-epoxy nanocomposites. Mater. Des..

[72] Vittayakorn W., Tepsansern P., Kriangkraikul W., Vittayakorn N. (2023). BaTiO3/Epoxy
Resin Nanocomposites as Flexible Energy Storage Devices. Integr. Ferroelectr..

