# Supplementary material for: Advanced Epoxy Polymer Composite Design: Maximizing Fracture Toughness and Piezoelectric Effect with Core–Shell Rubber and Barium Titanate Particles
Source: ACS Omega. 2025 Sep 26;10(39):45025–41. doi: 10.1021/acsomega.5c03542 (PMC12508967; doi:10.1021/acsomega.5c03542)
Supplement: Supplementary file 1 [file ao5c03542_si_001.pdf]

Advanced epoxy polymer composite design: Maximising fracture toughness and piezoelectric effect with core-shell rubber and barium titanate particles

Miray Yasar[a,b†], Berran Sanay[c], Cormac Duffy[c], Neal Murphy[a,b]*, Barry Burns[c], Alojz Ivankovic[a,b]

[a]University College Dublin, School of Mechanical and Materials Engineering, Dublin 4, Ireland

[b]I-Form Advanced Manufacturing Research Centre, Dublin 4, Ireland

[c]Henkel Ireland Operations & Research Ltd., Dublin, D24 YH42, Ireland

† Current Address: Luxembourg Institute of Science and Technology, Hautcharage, L-4940, Luxembourg

***Corresponding Author:**

Neal Murphy

E-mail address: neal.murphy@ucd.ie

Postal address: University College Dublin, School of Mechanical and Materials Engineering, Belfield, Dublin 4, Ireland

ORCID iDs

Miray Yasar https://orcid.org/0000-0002-6078-4201

Neal Murphy https://orcid.org/0000-0002-7236-0932

Alojz Ivankovic https://orcid.org/0000-0002-3938-828X

SUPPORTING INFORMATION

Dispersion of BT and CSR Particles in Epoxy Matrix

Since BT particles are in the micron size range while CSR particles are nanoscale, it is not possible to observe both particles within the same magnification. Therefore, the dispersion of BT particles was first evaluated, followed by analysis of CSR particles at higher magnifications.

1. **BT Dispersion in Lower Magnifications**

Table S1. % Area of barium titanate as a function of CSR content.

Formulation	BT Area % (Image-based)
CSR0	0.499
CSR5	1.121
CSR10	2.835
CSR15	2.669
CSR25	3.576

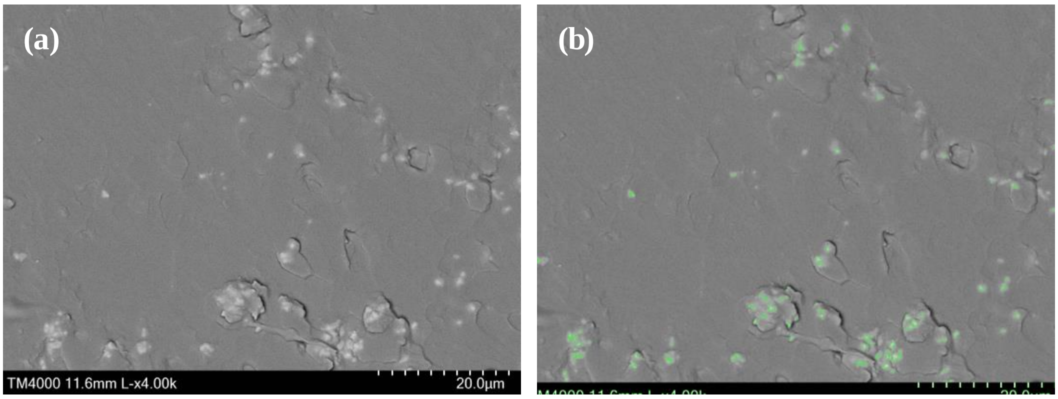

Figure S1. SEM micrographs of fracture surfaces of epoxy-based polymer composites containing 20 wt.% BT and 0 wt.% CSR (CSR0): (a) 20µm/ x4000 image, (b) Image J analysis for the BT dispersion.

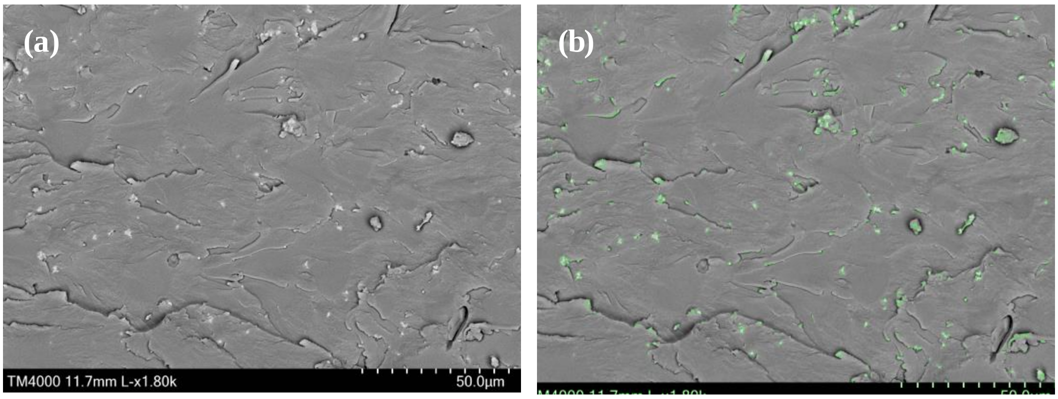

Figure S2. SEM micrographs of fracture surfaces of epoxy-based polymer composites containing 20 wt.% BT and 5 wt.% CSR (CSR5): (a) 20µm/ x4000 image, (b) Image J analysis for the BT dispersion.

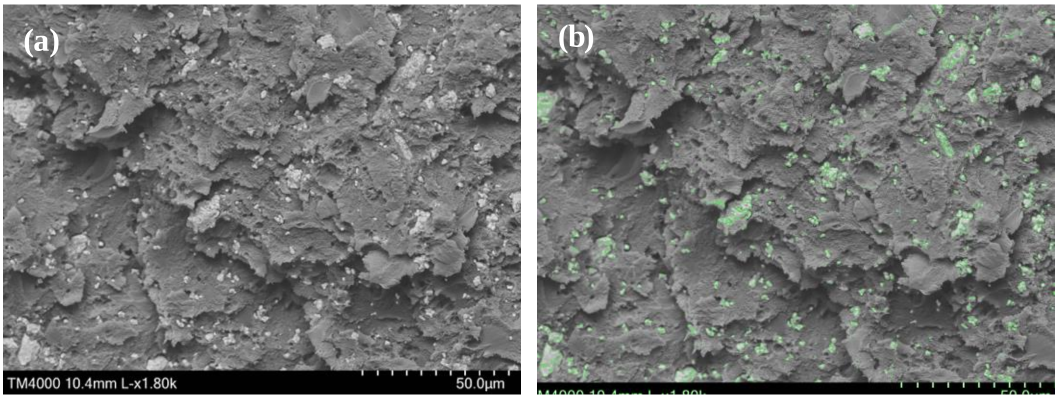

Figure S3. SEM micrographs of fracture surfaces of epoxy-based polymer composites containing 20 wt.% BT and 10 wt.% CSR (CSR10): (a) 20μm/ x4000 image, (b) Image J analysis for the BT dispersion.

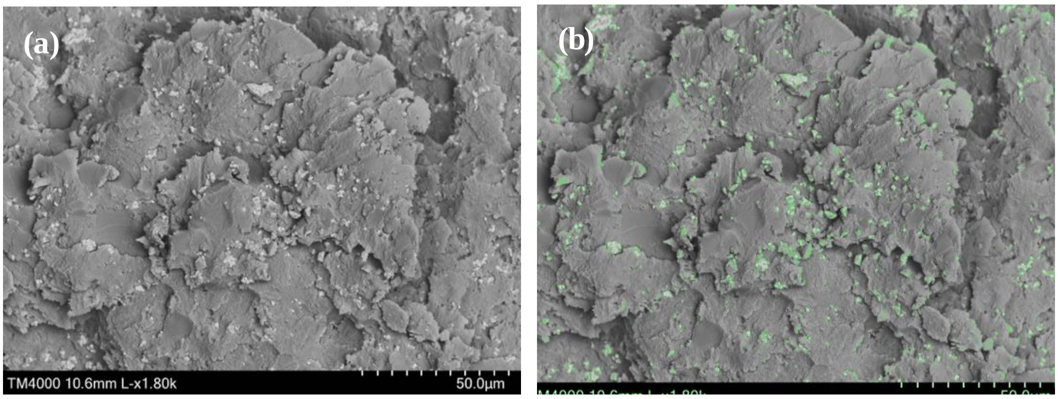

Figure S4. SEM micrographs of fracture surfaces of epoxy-based polymer composites containing 20 wt.% BT and 15 wt.% CSR (CSR15): (a) 20μm/ x4000 image, (b) Image J analysis for the BT dispersion.

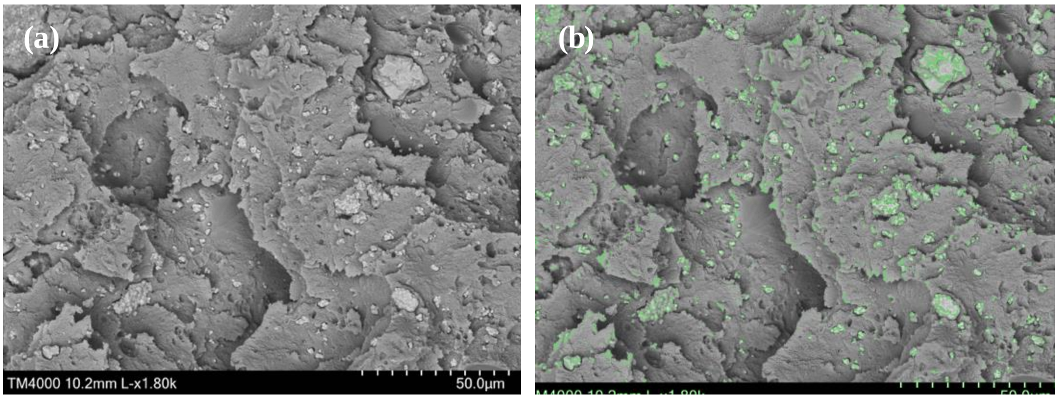

Figure S5. SEM micrographs of fracture surfaces of epoxy-based polymer composites containing 20 wt.% BT and 25 wt.% CSR (CSR25): (a) 20μm/ x4000 image, (b) Image J analysis for the BT dispersion.

2. CSR Dispersion in Higher Magnifications

Table S2. % Area of Core-shell rubber as a function of CSR content.

Formulation	CSR Count	Total CSR Area (µm²)	Image Area (µm²)	CSR Area %
CSR0	0	0	0	0%
CSR5	20	0.335	4.511	7.43%
CSR10	46	0.707	4.537	15.58%
CSR15	56	0.821	4.521	18.16%
CSR25	59	0.936	4.546	20.59%

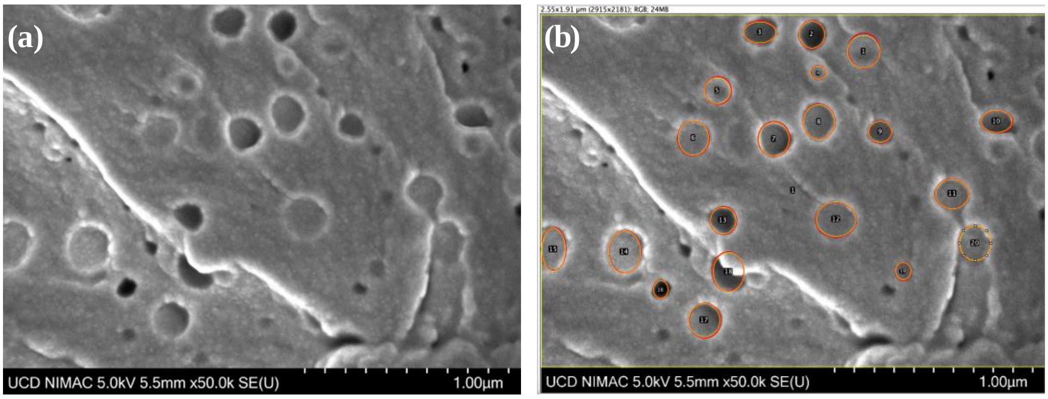

Figure S6. SEM micrographs of fracture surfaces of epoxy-based polymer composites containing 20 wt.% BT and 5 wt.% CSR (CSR5): (a) 1µm/ x50000 image, (b) Image J analysis for the CSR dispersion.

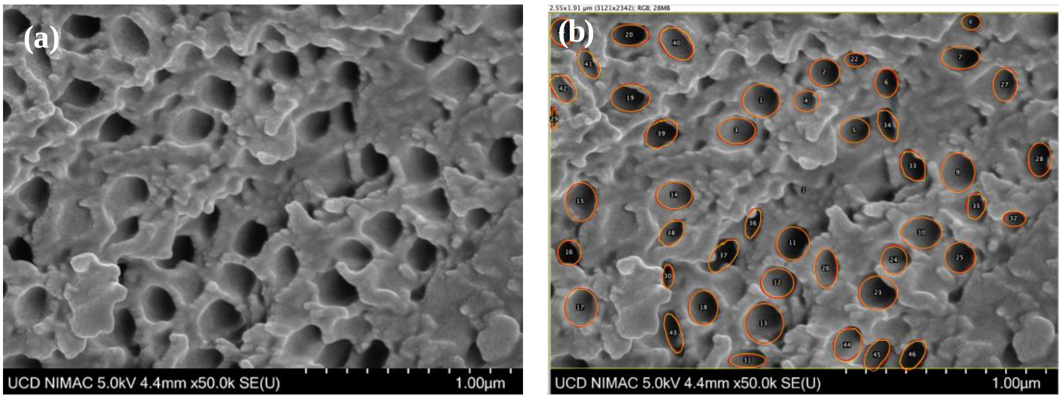

Figure S7. SEM micrographs of fracture surfaces of epoxy-based polymer composites containing 20 wt.% BT and 10 wt.% CSR (CSR10): (a) 1µm/ x50000 image, (b) Image J analysis for the CSR dispersion.

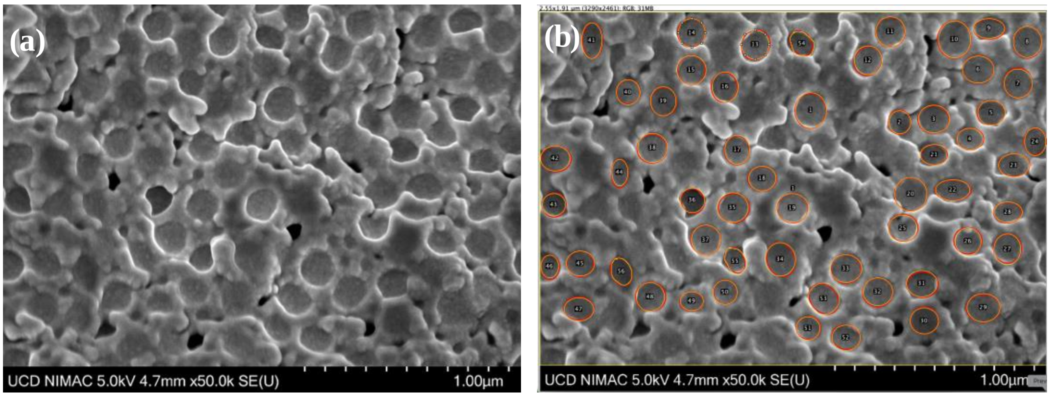

Figure S8. SEM micrographs of fracture surfaces of epoxy-based polymer composites containing 20 wt.% BT and 15 wt.% CSR (CSR15): (a) 1μm/ x50000 image, (b) Image J analysis for the CSR dispersion.

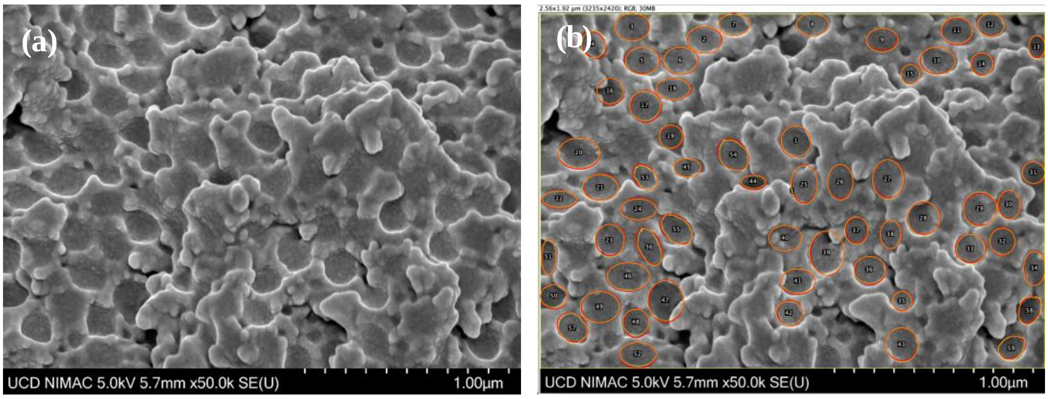

Figure S9. SEM micrographs of fracture surfaces of epoxy-based polymer composites containing 20 wt.% BT and 25 wt.% CSR (CSR25): (a) 1μm/ x50000 image, (b) Image J analysis for the CSR dispersion.
